# *Mgp* High‐Expressing MSCs Orchestrate the Osteoimmune Microenvironment of Collagen/Nanohydroxyapatite‐Mediated Bone Regeneration

**DOI:** 10.1002/advs.202308986

**Published:** 2024-04-08

**Authors:** Zhuqing Wan, Xiaoqiang Bai, Xin Wang, Xiaodong Guo, Xu Wang, Mo Zhai, Yang Fu, Yunsong Liu, Ping Zhang, Xiao Zhang, Ruili Yang, Yan Liu, Longwei Lv, Yongsheng Zhou

**Affiliations:** ^1^ Department of Prosthodontics Peking University School and Hospital of Stomatology Haidian District Beijing 100081 China; ^2^ National Center for Stomatology, National Clinical Research Center for Oral Disease, National Engineering Research Center of Oral Biomaterials and Digital Medical Devices, Beijing Key Laboratory of Digital Stomatology, NHC Key Laboratory of Digital Stomatology, Key Laboratory of Digital Stomatology Chinese Academy of Medical Sciences Haidian District Beijing 100081 China; ^3^ Department of Orthodontics Peking University School and Hospital of Stomatology Haidian District Beijing 100081 China

**Keywords:** biomaterials, mesenchymal stem cells, osteoimmune microenvironment, single‐cell transcriptomics, spatial transcriptomics

## Abstract

Activating autologous stem cells after the implantation of biomaterials is an important process to initiate bone regeneration. Although several studies have demonstrated the mechanism of biomaterial‐mediated bone regeneration, a comprehensive single‐cell level transcriptomic map revealing the influence of biomaterials on regulating the temporal and spatial expression patterns of mesenchymal stem cells (MSCs) is still lacking. Herein, the osteoimmune microenvironment is depicted around the classical collagen/nanohydroxyapatite‐based bone repair materials via combining analysis of single‐cell RNA sequencing and spatial transcriptomics. A group of functional MSCs with high expression of matrix Gla protein (*Mgp*) is identified, which may serve as a pioneer subpopulation involved in bone repair. Remarkably, these *Mgp* high‐expressing MSCs (*Mgp* ^hi^MSCs) exhibit efficient osteogenic differentiation potential and orchestrate the osteoimmune microenvironment around implanted biomaterials, rewiring the polarization and osteoclastic differentiation of macrophages through the *Mdk/Lrp1* ligand–receptor pair. The inhibition of *Mdk/Lrp1* activates the pro‐inflammatory programs of macrophages and osteoclastogenesis. Meanwhile, multiple immune‐cell subsets also exhibit close crosstalk between *Mgp* ^hi^MSCs via the secreted phosphoprotein 1 (SPP1) signaling pathway. These cellular profiles and interactions characterized in this study can broaden the understanding of the functional MSC subpopulations at the early stage of biomaterial‐mediated bone regeneration and provide the basis for materials‐designed strategies that target osteoimmune modulation.

## Graphical abstract

This study reveals how collagen/nanohydroxyapatite‐based bone repair materials regulate the osteoimmune microenvironment via combining single‐cell RNA sequencing and spatial transcriptomics analyses. The *Mgp* high‐expressing MSCs with efficient osteogenic differentiation potential are found to be aggregated around implanted materials at the early stage of bone regeneration, regulating the polarization and osteoclastic differentiation of macrophages through the *Mdk/Lrp1* ligand–receptor pair.

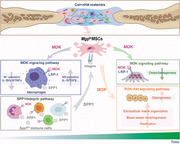

## 1 Introduction

Bone defects suffering from trauma, infections, tumors, or congenital disorders still present a major global health concern.^[^ ^1^ ^]^ With the rapid development of biomaterials and in‐depth research of stem cells, it is possible to achieve bone regeneration through tissue engineering technology.^[^ ^2^ ^]^ Generally, stem cells, biomaterials, and growth factors are the three major elements of bone tissue engineering.^[^ ^3^ ^]^ Nevertheless, inadequate separation and expansion efficiency, unequal in vitro differentiation potential, as well as safety issues of stem cells hampered their further clinical translations.^[^ ^4^ ^]^ Uncovering the cellular and molecular mechanisms of biomaterials interacting with autologous stem cells is more alluring to devise efficient strategies for bone regeneration.^[^ ^5^ ^]^ Meanwhile, the bone repair microenvironment is composed of heterogeneous cell populations including mesenchymal stem cells (MSCs) and immune cells with complex phenotypes and different functions.^[^ ^6^ ^]^ It is necessary to reveal the spatiotemporal response of functional subsets for enhanced bone regeneration at a high resolution, which could serve as the theoretical foundation for the development of efficient bone repair materials.

Over the past decade, the ongoing technological revolution has enabled to definition of the gene expression patterns of single cells and facilitates to dissecting of relevant cellular mechanisms that were previously hidden.^[^ ^7^ ^]^ In particular, single‐cell RNA‐sequencing (scRNA‐seq) has been developed to classify the cell heterogeneity, lineage tracing, and functions within the osteoimmune microenvironment for bone homeostasis or disease in vivo at a high resolution.^[^ ^8^ ^]^ To date, *LepR* ^+^ MSCs, *Pdgfra* ^+^ MSCs, and *Ctsk* ^+^ MSCs have been reported to play essential roles in bone development and bone healing.^[^ ^9^ ^]^ Besides, it has been reported that *Msx1* ^+^ skeletal stem cells could be recruited by neurotrophic supplements and *Krt14* ^+^ *Ctsk* ^+^ osteoprogenitors govern the maxillofacial bone homeostasis after maxillary sinus floor lifting surgery for enhanced bone regeneration, while their immunoregulatory functions have not been explored.^[^ ^10^ ^]^

Additionally, despite the cellular marker of functional MSCs, their spatial distributions around biomaterials are still poorly investigated. Spatial transcriptomics (ST) technology has been developed to elucidate the cellular and spatial heterogeneity within the area of interest by positioning histological sections on arrayed reverse transcription primers with unique positional barcodes.^[^ ^11^ ^]^ Taken together, the combination of scRNA‐seq and ST technologies could provide a comprehensive spatiotemporal landscape of the characteristic distribution and gene expression patterns of MSC subsets and immune cells, offering a broadly applicable strategy to systemically map the whole microenvironment around implanted biomaterials in vivo.^[^ ^12^ ^]^

In this study, the classical collagen/nanohydroxyapatite (nHA)‐based bone repair materials, which are basic components of natural bone tissue and have exhibited great potential for clinical translation, were utilized to depict the spatiotemporal transcriptomics map of microenvironment at the bone defect region after implantation of biomaterials.^[^ ^13^ ^]^ The cellular heterogeneity, physiological functions of MSCs and immune cells as well as their communication at the early stage of bone regeneration were comprehensively characterized via the combined analysis of scRNA‐seq and ST technologies. Remarkably, an MSCs subpopulation with a high expression level of matrix Gla protein (*Mgp*) was detected with significantly increased migration to the bone defect regions and progressively participated at the early stage of biomaterial‐mediated bone regeneration. They also had close cell–cell interactions with macrophages and performed potential immunomodulatory properties, which could mitigate the local inflammatory response. This study further deepens our understanding of the single‐cell spatiotemporal characterization of autologous cells regulated by implanted biomaterials, and the obtained results are expected to improve and optimize the design of cell‐targeted bone repair materials.

## 2 Results

### 2.1 The Bioactive Collagen/Nanohydroxyapatite Hydrogel Composites Enhanced New Bone Formation in Calvarial Defects

**Figure 1:**
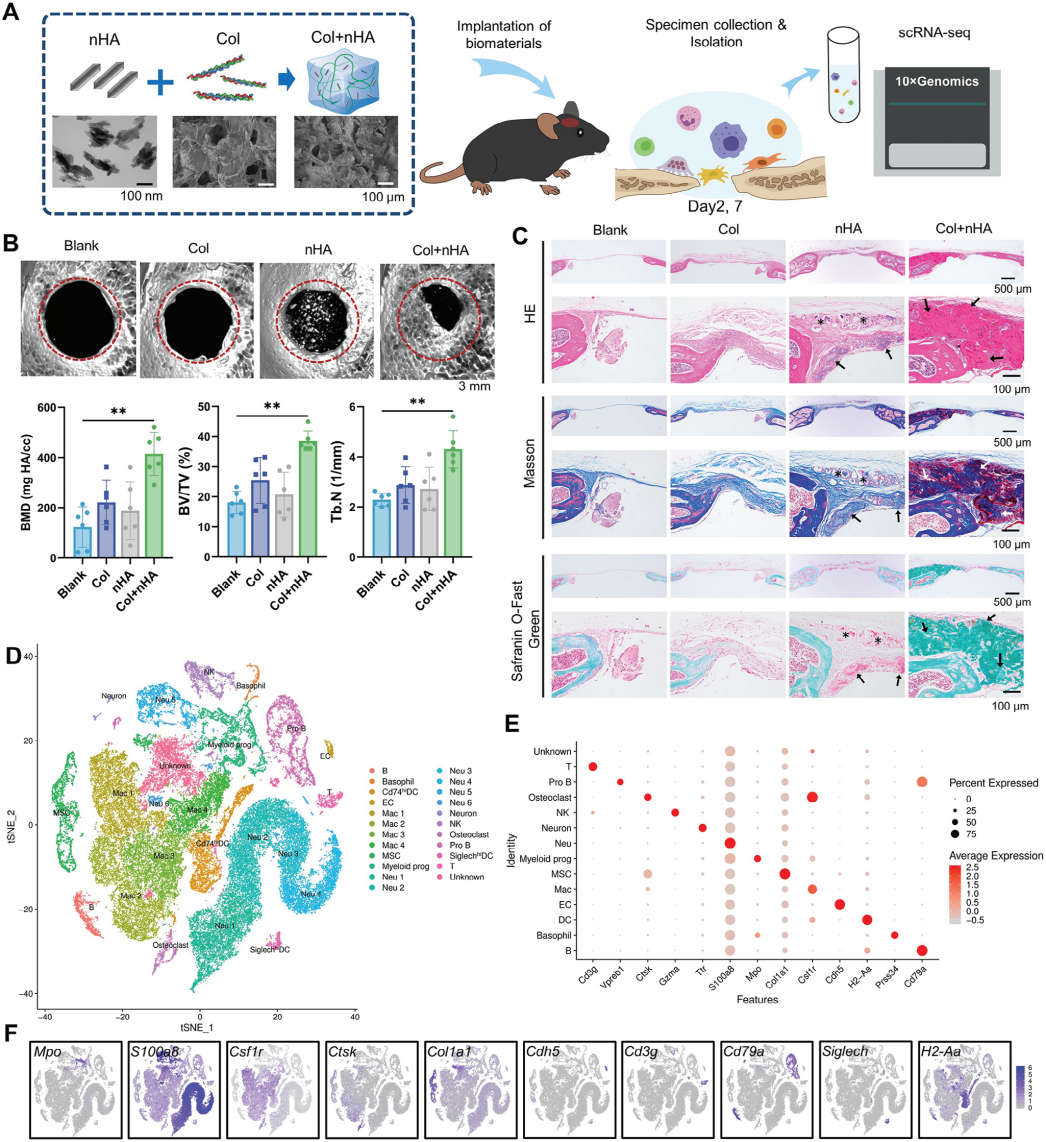
Expression landscape of mice calvarial bone defects repaired with or without bioactive Col+nHA hydrogel composites by scRNA‐seq. A) Schematic illustration for fabrication of Col+nHA hydrogel composites and a diagrammatic sketch of the scRNA‐seq analysis. B) Micro‐CT evaluation and quantitative analysis of bone regeneration in defect areas after 12 weeks’ observation (*n* = 6). BMD: Bone mineral density, BV/TV: bone volume/tissue volume, Tb.N.: trabecular number. C) Histological analysis of calvarial defects 12 weeks after implantation. Cross‐sections were stained with hematoxylin and eosin, Masson's trichrome, and Safranin O‐fast green to observe new bone formation. *: undegradable nHA particles; arrows: the newly formed bone tissue. D) Cells identified by scRNA‐seq were visualized with a *t*‐distributed stochastic neighbor embedding (t‐SNE) plot. Different cell populations were defined and distinguished by color. B: B cell. DC: dendritic cell. EC: endothelial cell. Mac: macrophage. MSC: mesenchymal stem cell. Myeloid prog: myeloid progenitor. Neu: neutrophil. NK: natural killer cell. Pro B: Pro B cells. T: T cells. E) Specific expression of marker genes in different cell types. F) The expression levels of marker genes were projected onto the t‐SNE atlas.

Collagen and nHA are two major constituents of natural bone tissue. Herein, a bioactive hydrogel composite was fabricated via incorporating nHA with type I collagen (Col). Transmission electron microscope (TEM) analysis showed that nHA particles were 80–100 nm in length and 15–20 nm in width (**Figure** **1**A), which is similar to the hydroxyapatite nanocrystals in natural bone tissue (≈40–60 nm long, 20 nm wide).^[^ ^14^ ^]^ The scanning electron microscopy (SEM) images showed that Col and Col+nHA hydrogels exhibited porous structures with good connectivity (Figure 1A). The Col+nHA hydrogel composite embedded with nHA particles had a looser internal micro‐structure with an average pore size ≈200 µm. The micro‐computed tomography (micro‐CT) reconstructions after 12 weeks of observation indicated that Col+nHA hydrogel composite assisted bone regeneration (Figure 1B). The quantitative analysis of new bone formation further showed a significantly higher bone mineral density and bone volume in the Col+nHA group compared with the Blank group. Consistently, the histological analysis revealed that the implanted materials were almost completely degraded after 12 weeks of observation in both Col and Col+nHA groups (Figure 1C). The implantation of Col+nHA resulted in significantly more new bone formation, while the majority of the defect region was filled with fibrous connective tissue, and few bone fragments were formed in the Blank and Col groups. The nHA group exhibited some immature bone tissue and undegraded nHA particles, which also could be observed in micro‐CT images. In a word, this typical Col+nHA hydrogel composite has a reliable capability to repair bone defects.

### 2.2 Single‐Cell Landscape of Mice Calvarial Bone Defects Repaired with or without Biomaterials

To investigate the biological reactions induced by bone repair materials at the early stage of bone healing, the regenerative tissue in calvarial defect regions repaired with or without Col+nHA hydrogel composites were dissected for enzymatic digestion and subjected to scRNA‐seq (Figure 1D). A total number of 65722 cells were obtained. We preprocessed the dataset with the Seurat package. Uniform Manifold Approximation and Projection (UMAP) and t‐distributed Stochastic Neighbor Embedding (t‐SNE) were calculated to visualize cell heterogeneity in reduced dimensions. As shown in Figure 1E, cells were divided into 14 clusters based on classic cell surface markers. According to these marker genes, these clusters were annotated as myeloid progenitors (*Mpo*); neutrophils (*S100a8*); macrophages (*Csfr1*); MSCs (*Col1a1*); endothelial cells (*Cdh5*); osteoclasts (*Ctsk*); T cells (*Cd3g*); B cells (*Cd79a*); pro B cells (*Vpreb1*); two clusters of dendritic cells (DC, *H2‐Aa*), expressing higher levels of *Cd74* and *Siglech*; natural killer cells (NK, *Gzma*); basophils (*Prss34*); neuron (*Ttr*) (Figure 1F). The top three expressed genes of each cluster were identified and compared in Figure S1A (Supporting Information). As immune cells and MSCs are highly plastic and can be recruited and polarized to different states depending on the microenvironment, we analyzed the cell ratios of the above clusters among defect areas repaired with or without hydrogel composites (Figure S1B,C, Supporting Information). The MSCs from the natural healing condition accounted for only 32.6% of the total cells, while 67.4% of MSCs were from samples implanted with hydrogel composites. The ratio of macrophages, NK cells, *Cd74* ^hi^ DC cells, and osteoclasts was also higher in samples implanted with bioactive materials, and the proportion of neutrophils was higher in blank samples. The proportion of macrophages and neutrophils was higher on day 2 compared with day 7, which was consistent with the time course of innate immune response (Figure S1D,E, Supporting Information). These results reflected enhanced immunoregulatory and bone remodeling processes during the biomaterial‐mediated bone regeneration.

**Figure 2:**
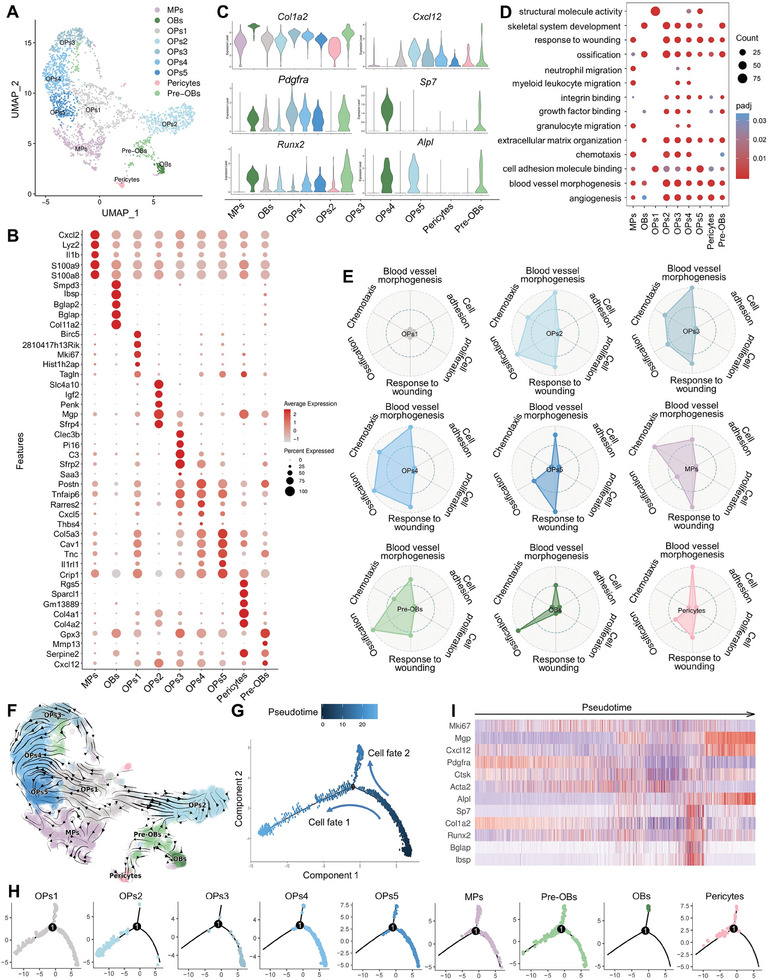
Heterogeneity of MSCs at the early stage of bone regeneration. A) Visualization of MSCs in Figure 1 with UMAP plot, which was divided into nine subclusters. MPs: myeloid progenitors. OPs: osteoprogenitors. OBs: osteoblasts. Pre‐OBs: pre‐osteoblasts. B) Dot plot showing the differentially expressed genes (DEGs) of different cell types. C) Violin plots showing the log‐normalized expression levels of curated feature genes in nine subclusters of MSCs. D) Enriched gene ontology (GO) terms of DEGs among the nine MSCs subcluster. E) Radar map showing the indicated function and metabolic pathway among each MSC subcluster. F) RNA velocity of MSCs estimated from unspliced and spliced transcripts of nearby cells and visualized on UMAP plot. G) Pseudotime lineage trajectory analysis indicating the direction of pseudotime and H) demonstrating the relationships of MSCs subclusters, color‐coded by subclusters. I) The expression dynamics of proliferative and osteogenic‐related genes in pseudotime.

Since the MSCs have been defined as multipotent stem cells and play essential roles during the process of bone regeneration, we subsequently distinguished MSCs into nine major subsets (**Figure** **2**A). These subsets were named based on the reported marker genes of stem cells: five subsets of osteoprogenitors (OPs) expressing *Cxcl12*, *Col1a2*, *Pdgfra*, and *Runx2*; pre‐osteoblasts (pre‐OBs) expressing *Sp7*, *Alpl*, *Bglap*; osteoblasts (OBs) with highly expressing abundance of osteogenic genes *Runx2*, *Alp*, *Sp7*, *Bglap* and hardly expressing *Cxcl12*; pericytes, expressing *Rgs5*, *Emcn*, *Col4a1*; myeloid progenitors (MPs), expressing *S100a8*, *S100a9*, *Cxcl2* (Figure 2B,C).^[^ ^12^, ^15^ ^]^

Gene ontology (GO) enrichment analysis revealed distinct functions of MSC subsets related to cell adhesion, cell proliferation, response to wounding, chemotaxis, blood vessel morphogenesis, and ossification, which were evaluated using a gene set (Figure 2D,E). The results showed that OPs2, OPs3, OPs4, Pre‐OBs, and OBs cells were featured by skeletal system development and ossification, and played essential roles in response to wounding. Based on the RNA velocity and pseudotime analysis, we uncovered the osteogenic differentiation trajectory of MSC subsets, originating from the OPs1 cells and then split into two main branches toward OPs2 and OBs (Figure 2F–H; Figure S2, Supporting Information). The patterns of proliferative and osteogenic biomarkers on the trajectory axis were shown as control (Figure 2I). OBs were found at the terminal end of the trajectory (cell fate 2 branch) with high expression of *Bglap* and *Ibsp*. Unlike the OBs subset, the OPs2 subset located at the terminal end of cell fate 1 branch highly expressed *Cxcl12* and *Alpl*, showing a high transcriptomic similarity to previously described osteo‐CAR (CXCL12‐abundant reticular) cells.^[^ ^15b^ ^]^

### 2.3 The Combined Analysis of scRNA‐Seq and ST‐Seq Revealed that OPs2 (*Mgp* ^hi^MSCs) Play Essential Roles at the Early Stage of Bone Regeneration

**Figure 3:**
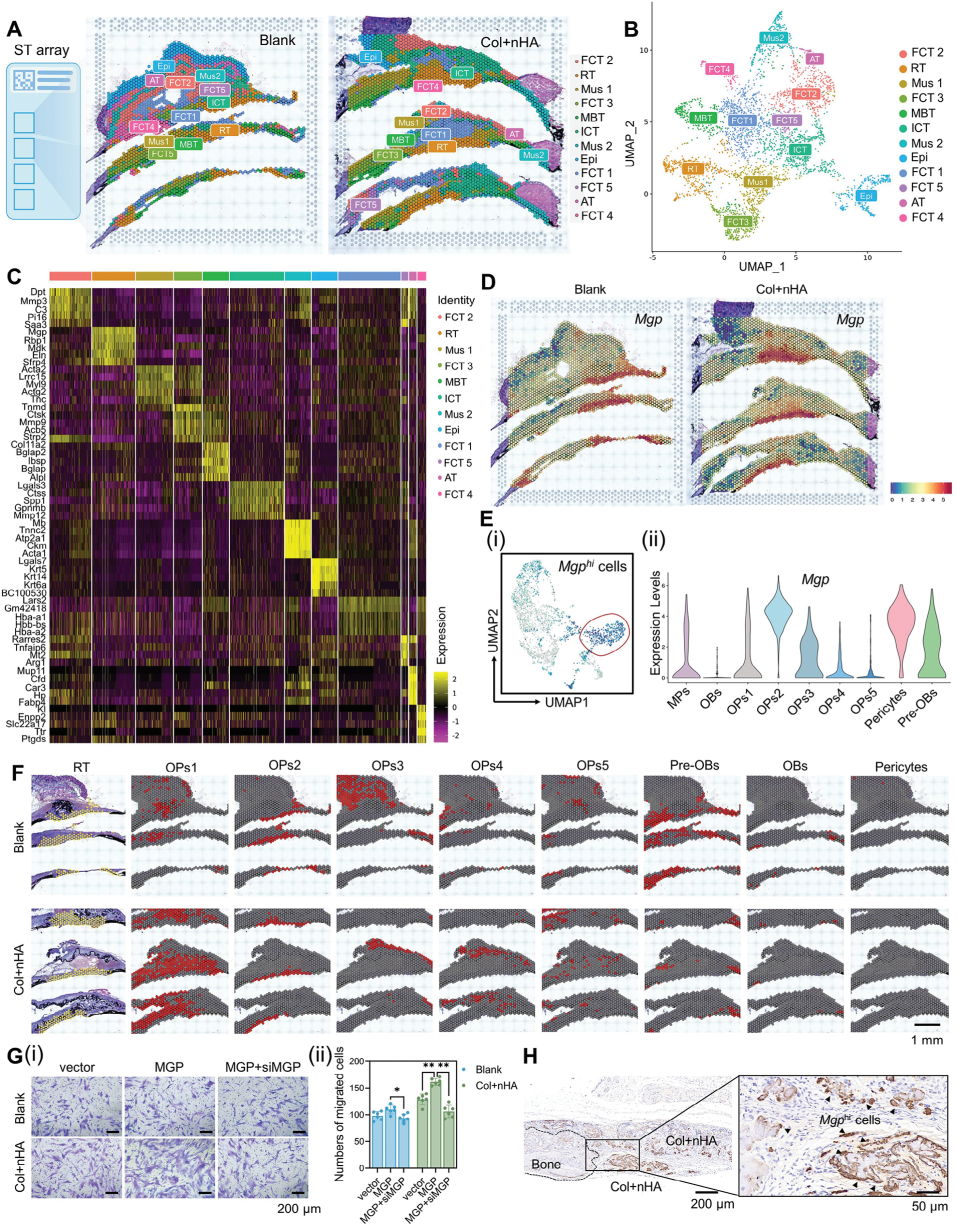
Tracing the spatial distribution of cell types within microenvironment of bone regeneration. A) The unsupervised clustering indicated the calvarial bone defects were repaired with or without bioactive Col+nHA hydrogel composites. Clusters with the same annotation are merged and assigned the same color code. FCT: fibrous connective tissue. RT: regenerative tissue. Mus: muscle. MBT: mature bone tissue. ICT: inflammatory connective tissue. Epi: epidermis. AT: adipose tissue. B) UMAP plot performed on the gene expression data from spots covered by tissue. K‐means clustering analysis has identified 12 types of spots, which have been assigned a color each. C) Heatmap showing the differentially expressed genes (DEGs) of different spot types. D) Spatial feature plots showed the normalized expression of *Mgp* in tissue sections. E) UMAP and violin plots showing the log‐normalized expression levels of *Mgp* in MSCs subsets. F) The spatial feature plot showed the locations of the ST cluster and defined subclusters of MSCs in tissue sections. G) Crystal violet staining showed the migrated hBMMSCs transfected with vector, hBMMSCs transfected with *MGP*‐overexpressing lentivirus (MGP), as well as hBMMSCs transfected with *MGP*‐overexpressing lentivirus and then interfered with siMGP‐1 (MGP+siMGP). The numbers of migrated cells per microscopic field were counted via Image J software (*n* = 6, **: *p *< 0.01.). H) Immunohistochemistry staining of *Mgp* ^hi^ cells around calvarial defect regions after 7 days of hydrogel implantation.

To explore the spatial characteristics of cell types during the process of bone regeneration, we applied ST‐seq to compare the spatial gene expression profiles between bone defect regions repaired with or without bioactive materials. After hematoxylin‐and‐eosin (HE) staining and brightfield imaging, the bone slides were subjected to distinguish the anatomical features, and a total of 4373 spatially barcoded spots were captured in two groups (**Figure** **3**A; Figure S3, Supporting Information). Standard quality control and dimensionality reduction were performed using Seurat methods, and visualization was realized through UMAP (Figure 3B). The clusters were annotated based on their histological features and differentially expressed genes (DEGs), including fibrous connective tissue (FCT), regenerative tissue (RT), muscle (Mus), mature bone tissue (MBT), inflammatory connective tissue (ICT), epidermis (Epi), as well as adipose tissue (AT) (Figure S4A, Supporting Information). Especially, the RT cluster also showed a higher expression level of *Mgp*, the marker gene of the OPs2 subset, as compared to other clusters (Figure 3C,D). The GO and Reactome analysis revealed that MBT, RT, Mus1, and FCT3 clusters expressed higher enrichment levels of extracellular matrix organization and collagen formation, responding to tissue damage (Figure S4B, Supporting Information). The RT cluster in the Col+nHA group activated more immune responses, especially innate immune responses, compared with the blank group, positively regulated the cell migration, enhancing the antigen processing and presentation, mediating the extracellular matrix organization process (Figure S4C, Supporting Information).

Furthermore, the combined analysis of scRNA‐seq and ST‐seq databases was performed to distinguish the functional MSC subcluster in RT. *Mgp*, the marker gene of the RT cluster in the ST database, was identified to be OPs2‐specific after calculating DEGs by comparing OPs2 to the rest of MSC subsets (aveLog_2_FC = 2.331, *p *= 5.011e−215). While, the expression distribution diagram of the MSCs showed that *Mgp* was highly expressed in the subsets of OPs2, pre‐OBs, partial cells of OPs1, and pericytes (Figure 3E). Then, we further mapped these MSC subclusters based on DEGs from the scRNA‐seq database to the calvarial defect tissue in the ST‐seq database. Scoring spots in each section showed that the OPs2 exhibited strong spatial preferences within the calvarial defect regions, while the other OPs subclusters exhibited a diffused distribution and the OBs cells located at the mature bone tissue (Figure 3F; Figure S3, Supporting Information). The pre‐OBs with high expression of *Cxcl12*, *Sp7*, and *Mmp13* were located around mature bone tissue and calvarial suture, rather than defect regions specifically. These results revealed that OPs2 cells could migrate to the defect regions at the early stage of bone defect repair and may play essential roles in tissue regeneration. Furthermore, we verified the in vitro chemotaxis of *Mgp* high‐expressing (*Mgp* ^hi^) MSCs by Col+nHA hydrogel composites. More *MGP*‐overexpressing hBMMSCs migrated to the lower chamber in the Col+nHA group after 24 h of culture compared with the hBMMSCs transfected with vector. Additionally, the *MGP*‐overexpressing hBMMSCs interfered with si*MGP* and exhibited reduced chemotaxis (Figure 3G). The immunohistochemistry (IHC) staining showed that abundant *Mgp* ^hi^ cells were aggregated within the bone‐defect regions and around implanted Col+nHA hydrogel composites in a 7‐day observation, which was consistent with the results of bioinformatics analysis (Figure 3H).

### 2.4 *MGP* was a Positive Regulator for the Osteogenic Differentiation of hBMMSCs

**Figure 4:**
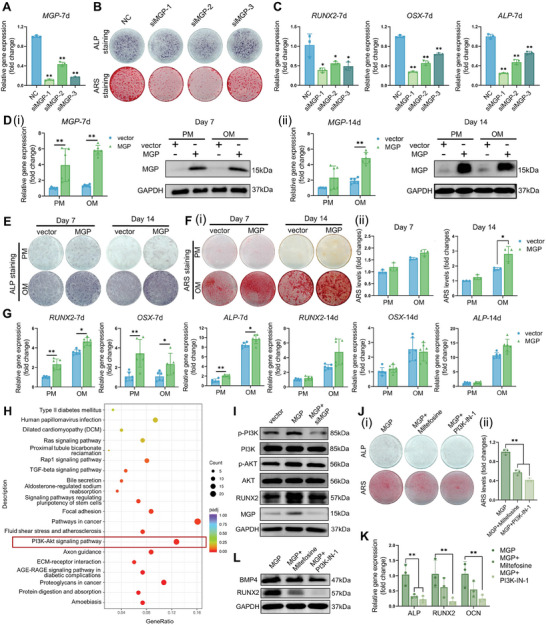
*MGP* overexpression enhances osteogenic differentiation of hBMMSCs in vitro. A) Relative mRNA expression levels of *MGP* in hBMMSCs transfected with si*MGP*‐1, si*MGP*‐2, si*MGP*‐3, and negative control (NC) siRNAs for 7 days. ALP/ARS staining of hBMMSCs with *MGP* knockdown B) and relative mRNA expression levels of osteogenic genes (*RUNX2*, *OSX*, and *ALP*) in hBMMSCs C) transfected with si*MGP*‐1, si*MGP*‐2, si*MGP*‐3, and NC siRNAs after 7 days of osteogenic induction. D) Efficiency of *MGP* overexpression was validated by RT‐qPCR and Western blot. ALP staining E), ARS staining and quantification analysis F) of *MGP*‐overexpressing hBMMSCs after 7 days and 14 days of incubation. G) *MGP* overexpression promoted the mRNA expression of *RUNX2*, *OSX*, and *ALP*. H) Upregulated Kyoto Encyclopedia of Genes and Genomes (KEGG) items of the OPs2 subset. I) MGP, RUNX2, AKT, p‐AKT, PI3K, and p‐PI3K protein expressions in the control group (vector), *MGP*‐overexpressing hBMMSCs transfected with or without si*MGP*‐1. J) ALP and ARS staining (i), ARS staining quantification analysis (ii) of *MGP*‐overexpressing hBMMSCs treated with inhibitors of PI3K‐Akt signaling pathway (Miltefosine and PI3K‐IN‐1). K) Relative expression levels of osteogenic genes. L) RUNX2 and BMP4 protein expressions in *MGP*‐overexpressing hBMMSCs treated with inhibitors. PM: proliferation medium; OM: osteogenic medium.

According to the results of scRNA‐seq and ST, we proposed that *Mgp* may play essential roles during the early stage of the MSC osteogenic differentiation process. Therefore, we explored the expression status of *MGP* in hBMMSCs after osteogenic induction at different time points (Figure S5A, Supporting Information). The results showed that the *MGP* expression level was elevated after culturing for 3 days and reduced after the next 7 days of culture. To further investigate the potential role of *MGP* in the osteogenic differentiation of MSCs, three small interfering RNA (siRNA) sequences were used to construct *MGP*‐deficient hBMMSCs (**Figure** **4**A). The downregulation of *MGP* decreased the ALP activity and formation of mineralized nodules in hBMMSCs, reducing the expressions of osteogenic genes after 7 days of osteogenic differentiation (Figure 4B,C). To confirm the role of *MGP* in the osteogenic differentiation of hBMMSCs, we constructed *MGP* stable overexpressing hBMMSCs, and the lentiviral transduction efficiency was confirmed by qRT‐PCR and western blot (Figure 4D). As shown in Figure 4E, ALP activity and mineralized matrix formation were significantly promoted in *MGP*‐overexpressing cells. The ARS quantification analysis indicated the same tendency (Figure 4F). In addition, the overexpression of *MGP* in hBMMSCs resulted in elevated expression levels of osteogenic markers, including *RUNX2*, *OSX*, and *ALP* (Figure 4G). Moreover, the mutant plasmids were also used to construct the *MGP*‐overexpressing cells. The results of staining and qRT‐PCR demonstrated the same tendency (Figure S5B,C, Supporting Information).

Furthermore, the GO enrichment and Kyoto Encyclopedia of Genes and Genomes (KEGG) pathway analysis suggested that genes assigned to the extracellular matrix, ossification, and PI3K‐Akt signaling pathway had significantly upregulated expression in the OPs2 subset (Figure 4H; Figure S5D, Supporting Information). Meanwhile, the *MGP*‐overexpressing hBMMSCs exhibited increased expression levels of RUNX2 and phosphorylation levels of AKT (p‐AKT) and PI3K (p‐PI3K), indicating that MGP may promote osteoblastic differentiation of MSCs via PI3K‐Akt signaling pathway (Figure 4I). In addition, the inhibition of *MGP* decreased the activation of p‐AKT and p‐PI3K. Furthermore, two kinds of inhibitors of the PI3K‐Akt signaling pathway, miltefosine, and PI3K‐IN‐1, were applied to evaluate the influence of inhibiting PI3K‐Akt signaling pathway on the osteogenic differentiation of *MGP*‐overexpressing hBMMSCs. The inhibitors' treatment significantly suppressed the osteogenic differentiation of *MGP*‐overexpressing hBMMSCs (Figure 4J–L). Collectively, our results demonstrated that *MGP* was a positive regulator for the osteogenic differentiation of MSCs in vitro.

### 2.5 MGP Could Modulate the Polarization and Osteoclastogenesis of Macrophages In Vitro

The early stage of bone regeneration, after injury and biomaterial implantation, involves a comprehensive physiological process between MSCs and immune cells to respond to tissue damage and orchestrate tissue regeneration. Since the *Mgp* ^hi^MSCs were abundant in defect regions at the early stage of the bone repair process and performed essential immunoregulatory functions in the Col+nHA group based on the differential functional analysis of ST‐seq (Figure 3; Figure S4C, Supporting Information), we further focused on the interactions between *Mgp* ^hi^MSCs and innate immune cells. The cellular composition of macrophages and osteoclast clusters were both obviously higher in Col+nHA group (71.39%, 73.07%) versus the Blank group (28.61%, 26.93%), while the cellular proportion of neutrophils was higher in the Blank group (63.33%), indicating the macrophages may serve as the main effector cells that respond to the stimulation of implant materials and the regulation of MSCs (Figure S1C, Supporting Information).

**Figure 5:**
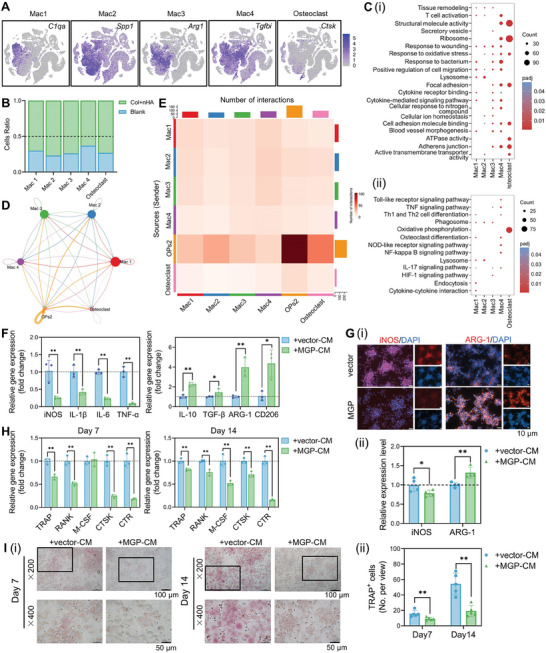
The cellular heterogeneity of macrophage/osteoclast subclusters and the function of MGP on macrophage polarization and osteoclastogenesis in vitro. A) t‐SNE atlas showing the expression levels of marker genes of macrophage subclusters and osteoclasts. B) Cell ratios of macrophage subclusters and osteoclasts in Blank and Col+nHA samples. C) Enriched GO and KEGG terms of DEGs among the seven macrophage/osteoclast clusters. Network diagram D) and heatmap E) showing the number of interactions between OPs2, macrophage, and osteoclast subclusters. F) Gene expression of M1 and M2‐related markers in human THP‐1 macrophages cultured with the conditional medium of *MGP*‐overexpressing hBMMSCs (MGP‐CM) and hBMMSCs transfected with empty vector (vector‐CM). G) Immunofluorescent (IF) staining and semi‐quantification showing the expression levels of iNOS and ARG1 in THP‐1 cells. Gene expression of osteoclastic markers H) and tartaric acidic phosphatase (TRAP) staining I) of THP‐1 cells cultured with vector‐CM or MGP‐CM.

Our scRNA‐seq dataset was classified into 23 clusters containing four clusters of macrophages (Mac1–4), which are characterized by different immune‐associated genes, including *C1qa*, *Spp1*, *Arg1*, and *Tgfbi*, respectively (**Figure** **5**A). Besides, a part of macrophages with high expression of osteoclastic genes (*Ctsk*, *Acp5*, and *Nrp2*) were identified as osteoclasts. As shown in Figure 5B, the cell ratios of four macrophage clusters and osteoclasts were obviously higher in samples repaired with Col+nHA hydrogel composites compared with the blank samples. The biological functions of Mac1–3 clusters were enriched in response to wounding, positive regulation of cell migration, and tissue remodeling, and Mac4 was related to T cell activation, structural molecule activity, ribosome, and adherens junction (Figure 5C). To reveal the differentiation dynamics of macrophages, we reconstructed the maturation trajectories analysis (Figure S6A, Supporting Information). Three clusters of macrophages (Mac1–3) were found at the original bifurcations of differentiation. Mac4 and osteoclasts were found near the terminal end of the trajectory (Figure S6B,C, Supporting Information).

Furthermore, we analyzed the interaction between the OPs2 subcluster and macrophages/osteoclasts via CellChat, a cell ligand/receptor pairing‐based database, based on the scRNA‐seq database (Figure 5D,E). The results indicated that the OPs2 subcluster may exhibit essential immunomodulatory effects and especially had a higher interaction with Mac2 and osteoclast subclusters. To further confirm the immunoregulatory function of MGP on macrophages in vitro, the human THP‐1 monocyte‐derived macrophages were cultured with the conditional medium of *MGP*‐overexpressing hBMMSCs (MGP‐CM) and hBMMSCs transfected with empty vector (vector‐CM). The gene expression of M1 markers, including *iNOS*, *IL‐6*, *IL‐1β*, and *TNF‐α*, was decreased in the MGP‐CM group after 4 days of induction, indicating that MGP may mitigate the inflammatory response (Figure 5F). Additionally, known M2‐related genes, including *IL10*, *ARG‐1*, and *CD206*, were expressed at higher levels in the MGP‐CM group. Furthermore, immunofluorescent staining against iNOS and ARG‐1 also exhibited the same trend (Figure 5G). The bulk RNA‐seq revealed the gene expression changes of macrophages (Figure S7A, Supporting Information). GO items, such as cell chemotaxis, chemokine‐mediated signaling pathway, and cytokine receptor binding, were significantly upregulated in the *MGP*‐overexpressing‐hBMMSCs‐cocultured group (Figure S7B, Supporting Information). KEGG pathway enrichment analysis reported that the cytokine–cytokine receptor interaction was upregulated and the cAMP signaling pathway, MAPK signaling pathway, and IL‐17 signaling pathway were downregulated (Figure S7C, Supporting Information). The above results suggested that MGP could influence macrophage polarization and regulate the local immune microenvironment via cytokine‐receptor interactions.

The RNA‐seq results also indicated that MGP could downregulate the osteoclastic differentiation of macrophages. The THP‐1‐derived macrophages can be further differentiated into mononuclear tartaric acidic phosphatase (TRAP)‐positive (TRAP^+^) preosteoclasts under the stimulation of M‐CSF and RANKL.^[^ ^16^ ^]^ We demonstrated that the expression levels of osteoclastic markers (i.e., *TRAP*, *RANK*, *M‐CSF*, *CTSK*, and *CTR*) were downregulated in the MPG‐CM group (Figure 5H). There was a significantly decreased TRAP staining intensity of preosteoclasts cultured with MGP‐CM (Figure 5I), indicating that MGP could migrate the osteoclastogenesis in vitro.

### 2.6 *Mgp* ^hi^MSCs Influenced the Functions of Macrophages via *Mdk/Lrp1* Ligand–Receptor Pair

**Figure 6:**
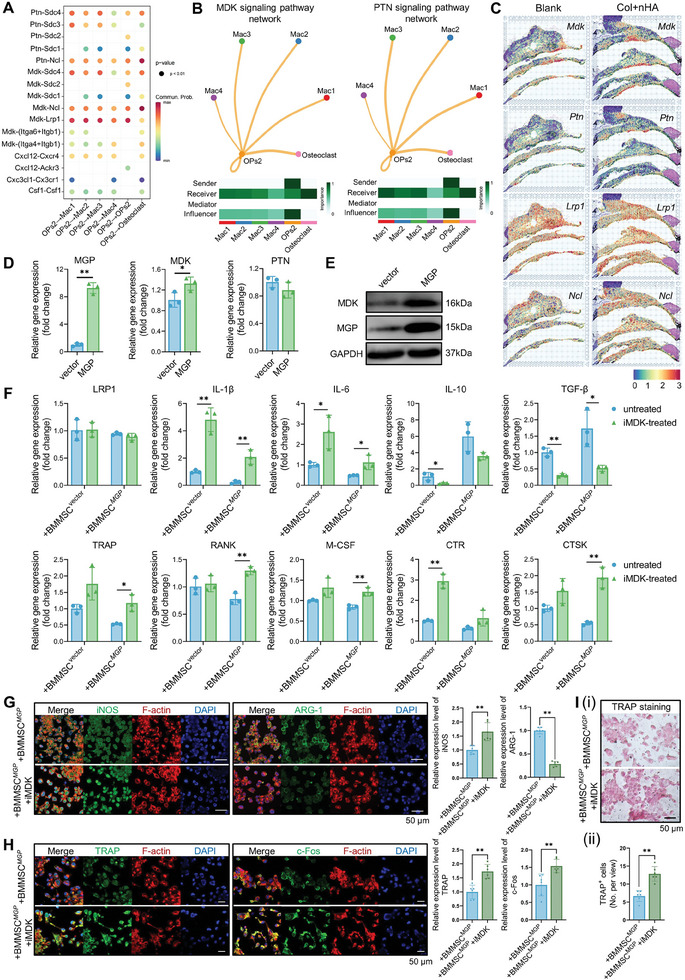
*Mgp* ^hi^MSCs influenced the functions of macrophages by activating the MDK signaling pathway. A) Unique ligand–receptor pairs between OPs2 (*Mgp* ^hi^MSCs), macrophage, and osteoclasts subclusters. B) Circle plots showing the inferred MDK, and PTN signaling networks between OPs2 and macrophages. C) Spatial feature plots showed the normalized expression of *Mdk*, *Lrp1*, *Ptn*, and *Ncl* in tissue sections. D) Relative expression of *MGP*, *MDK*, *PTN* in hBMMSCs. E) The protein expression level of MDK was upregulated in *MGP*‐overexpressing hBMMSCs. F) The MDK inhibitor (iMDK) treatment influences the expression levels of genes associated with inflammatory response and osteoclastogenesis. G) iMDK treatment influences the expression levels of M1/M2‐related markers (iNOS/ARG‐1) in THP‐1‐derived macrophages observed via a laser‐scanning confocal microscope. H) iMDK treatment influences the expression levels of osteoclastic markers (c‐Fos and TRAP) in THP‐1‐derived macrophages. I) iMDK treatment influences the TRAP staining of THP‐1 derived macrophages after 7 days of osteoclastic induction. +BMMSCs^vector^: THP‐1 derived macrophages cocultured with hBMMSCs transfected with lentiviral vector; +BMMSCs^MGP^: THP‐1 derived macrophages cocultured with *MGP*‐overexpressing hBMMSCs.

To investigate the underlying cellular mechanism of *Mgp* ^hi^ MSCs regulating the functions of macrophages, we applied CellChat analysis to identify ligand–receptor pairs among the OPs2 subcluster and macrophage/osteoclasts clusters (**Figure** **6**A; Figure S8, Supporting Information). The most specific interactions were observed with immunosuppression (such as TGFB, PTN, MDK, IGF, and CXCL12 signaling). Notably, the MDK (*Mdk/Ncl*, *Mdk/Lrp1*, *Mdk/Sdc4*) and PTN (*Ptn/Ncl*, *Ptn/Sdc4*) signaling pathways were most significantly enriched in MSC‐macrophage crosstalk in two groups at different time points. The Mac1 and Mac2 subclusters and osteoclasts were the main receivers of MDK signals, and OPs2 cells functioned as the senders of communication (Figure 6B). The Mac1 and osteoclast clusters were the leading receivers of the PTN signaling pathway. To further examine the anatomic relationship between OPs2 cells and macrophage/osteoclast clusters, the key cytokines involved in MSC‐macrophage crosstalk were plotted on tissue sections (Figure 6C). The expressions of *Mdk* and *Lrp1* were mainly located at the defect regions, suggesting they play essential roles in the process of bone defect healing.

Midkine (MDK) is categorized as a heparin‐binding protein and is associated with immune suppression and drug resistance in cancer.^[^ ^17^ ^]^ We found that the *MGP*‐overexpressing hBMMSCs had higher gene and protein expression levels of MDK compared with the negative control group (Figure 6D,E). To ensure the importance of MDK expression on immunoregulatory effects of *Mgp* ^hi^MSCs in vitro, we cocultured the THP‐1 derived macrophages with *MGP*‐overexpressing hBMMSCs (BMMSCs^MGP^) via Transwell system, then treated with DMSO and an MDK inhibitor (iMDK), which could specifically inhibit MDK but not influence the effect of other growth factors such as PTN (homologous to MDK).^[^ ^18^ ^]^ RT‐qPCR analysis showed that the iMDK treatment did not affect the gene expression level of its receptor, *LRP1*, while enhancing the expression of M1‐related genes (*IL‐6*, *IL‐1β*) and osteoclastic genes (*TRAP*, *RANK*, *M‐CSF*, *CTSK*, *CTR*), decreasing the expression of M2‐related genes (*IL‐10*, *TGF‐β*) (Figure 6F). The immunofluorescent staining and semi‐quantitative analysis against the M1‐related marker (iNOS) and M2‐related marker (ARG‐1) indicated the same trend after 4 days' coculture of THP‐1 derived macrophages and BMMSCs^MGP^ (Figure 6G). We also found that iMDK treatment significantly promoted the expression of osteoclastic markers (c‐Fos and TRAP), increasing the number of TRAP^+^ cells (Figure 6H,I). These results potentially indicated that MDK could reduce the pro‐inflammatory and osteoclastic phenotypes of macrophages and this effect would be counteracted by its inhibitor, iMDK.

**Figure 7:**
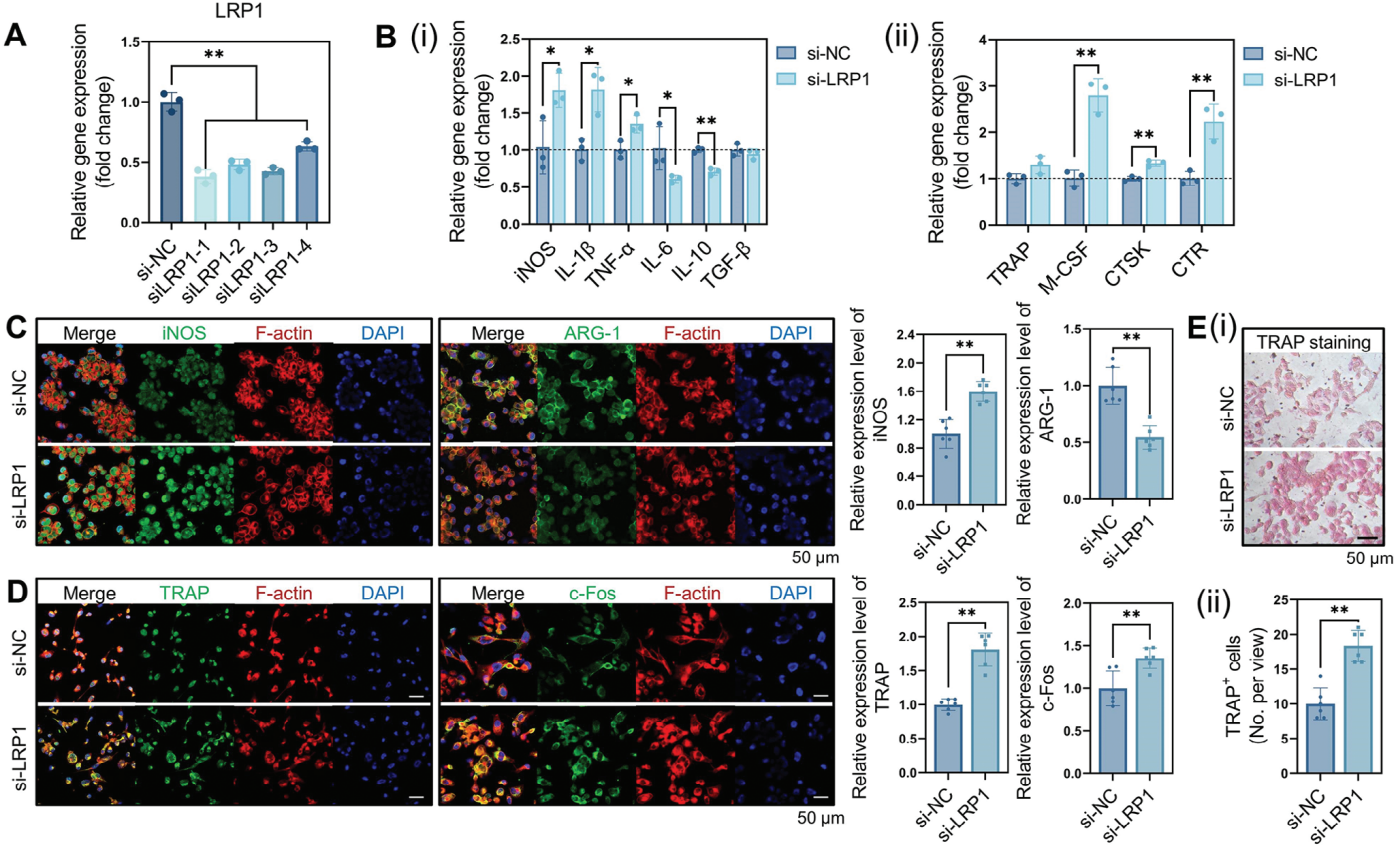
Inhibiting the expression level of the LRP1 receptor on macrophages increased their pro‐inflammatory differentiation and osteoclastogenesis. A) Relative mRNA expression levels of *LRP1* in THP‐1 derived macrophages transfected with si*LRP1*‐1, si*LRP1*‐2, si*LRP1*‐3, si*LRP1*‐4, and negative control (NC) siRNAs for 4 days. B) The expression levels of M1/M2‐related and osteoclastic genes in macrophages transfected with si*LRP1*‐1 and NC siRNA. C) The knock‐down of *LRP1* influenced the expression levels of iNOS/ARG‐1 and D)TRAP/c‐Fos. E) TRAP staining of macrophages with *LRP1* knock‐down.

To investigate whether *Mg*p^hi^MSCs could influence the functions of macrophages via the LDL receptor related protein 1 (LRP1), we transfected THP‐1‐derived macrophages with siRNAs to knock down the expression of the LRP1 receptor, then cultured them with a conditional medium of *MGP*‐overexpressing hBMMSCs. The transfection efficiency has been verified via PCR and IF staining (**Figure** **7**A; Figure S9, Supporting Information). The si*LRP1*‐1 sequencing was chosen for further investigation. The knock‐down of *LRP1* increased the pro‐inflammatory polarization and osteoclastic differentiation of macrophages, indicating that LRP1 may serve as a potential receptor target on macrophages against the regulatory function of MSCs (Figure 7B–E).

### 2.7 *Spp1* was Upregulated in Multiple Immune Cell Clusters Within the Osteoimmune Microenvironment Implanted with Bioactive Materials and Served as a Potential Therapeutic Regulator on *Mgp* ^hi^MSCs for Bone Regeneration

**Figure 8:**
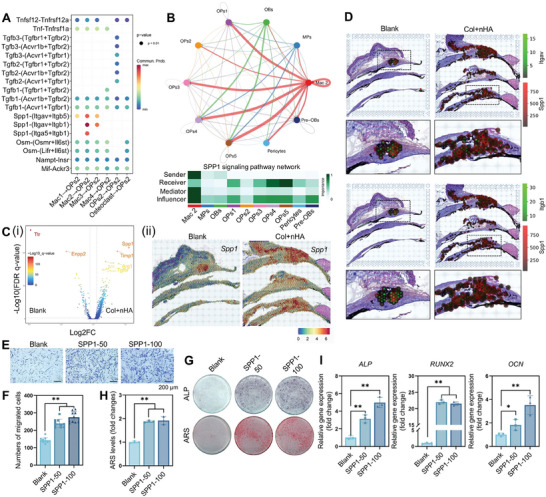
The *Spp1* ^hi^ cells regulated the functions of *Mgp* ^hi^MSCs (OPs2) via the *Spp1* signaling pathway. A) Unique ligand‐receptor pairs between macrophage subsets, osteoclast, and OPs2. B) Circle plots showing the inferred SPP1 signaling network between Mac2 and MSCs subclusters. C) Volcano plot showing the DEGs of RT cluster in (i) ST‐seq and the (ii) spatial feature plots showing the normalized expression of *Spp1* in Blank and Col+nHA samples. D) Spatial feature plots showing the cellular interactions via *Spp1/Itgav* and *Spp1/Itgb1* ligand–receptor pairs between spots in tissue sections. Red spots: *Spp1*; Green spots: *Itgav*/*Itgb1*. E) Transwell chemotaxis of hBMMSCs cultured with 50/100 ng mL^−1^ recombinant SPP1 protein. F) The quantitative analysis of the Transwell assay. ALP/ARS staining G), the quantitative analysis of ARS staining H), and the expression levels of osteogenic genes I) of hBMMSCs cultured with different concentrations of SPP1. SPP1‐50: 50 ng mL^−1^ recombinant SPP1 protein; SPP1‐100: 100 ng mL^−1^ recombinant SPP1 protein.

To evaluate the effect of implanted materials on the phenotype of macrophages, we compared the DEGs of macrophage subclusters in the Col+nHA group compared with the blank group, in which the *Ngp* was downregulated in all macrophage subclusters and *Spp1* was upregulated in Mac2, *Lgals3* was upregulated in Mac3, *Rps2* was upregulated in Mac4 (Figure S10, Supporting Information). To further clarify the regulatory functions of macrophages on the OPs2 subset, we plotted the essential ligand/receptor pairs involved in macrophage‐MSC crosstalk, including *Tgfb1*, *Spp1*, *Osm*, *Nampt*, and *Mif*‐related pathways (**Figure** **8**A). Specially, the Mac2 subcluster has a strong interaction with OPs2 subcluster via SPP1/Integrin pathway (*Spp1/(Itgav+Itgb5)*, *Spp1/(Itgav+Itgb1)*, *Spp1/(Itga5+Itgb1)* ligand–receptor pairs). Meanwhile, the Mac2 also exhibited close interactions with other MSC subclusters via the SPP1 signaling pathway (Figure 8B).

Remarkably, other immune cells, such as neutrophils, pro‐B cells, B cells, T cells, NK cells, and basophils, in biomaterial‐mediated bone repairing microenvironment showed significantly higher expression levels of *Spp1* compared with the natural healing microenvironment according to the volcano plot (Figure S11A, Supporting Information). To seek the critical factors of cellular interactions, we investigated the signaling networks between the above immune cells and the OPs2 subcluster (Figure S11B,C, Supporting Information). CellChat analysis revealed that the OPs2 subcluster exhibited close interactions with multiple immune cells via *Mdk/Ncl*, confirming the promising immunoregulatory roles of OPs2 and MDK (Figure S11D, Supporting Information). SPP1/Integrin pathway was also involved most among multiple immune cells and OPs2 (Figure S11E, Supporting Information). These results were also confirmed in the ST‐seq analysis. The upregulated expression of *Spp1* was detected in the RT cluster of the Col+nHA group compared with the Blank group (Figure 8C). The spatial distribution patterns of *Spp1/Itgav* and *Spp1/Itgb1* ligand–receptor pairs were enriched in Col+nHA samples, indicating the potential regulatory effects of *Spp1* ^hi^ cells on MSCs (Figure 8D).

Previous studies have reported the essential regulatory roles of secreted phosphoprotein 1 (SPP1/osteopontin) as an osteogenic differentiation‐related and immunoregulatory factor in bone remodeling and pathogenesis of various inflammatory diseases.^[^ ^19^ ^]^ Our results also confirmed the enhanced chemotaxis and osteogenic capabilities of SPP1 on hBMMSCs via using recombinant SPP1 protein (Figure 8E–I). In summary, *Spp1* was widely upregulated in multiple immune cell clusters in Col+nHA group, playing critical roles in remodeling the immune microenvironment during the biomaterial‐mediated bone repair process and serving as the potential regulator for the design of immunoregulatory materials.

## 3 Discussion

**Figure 9:**
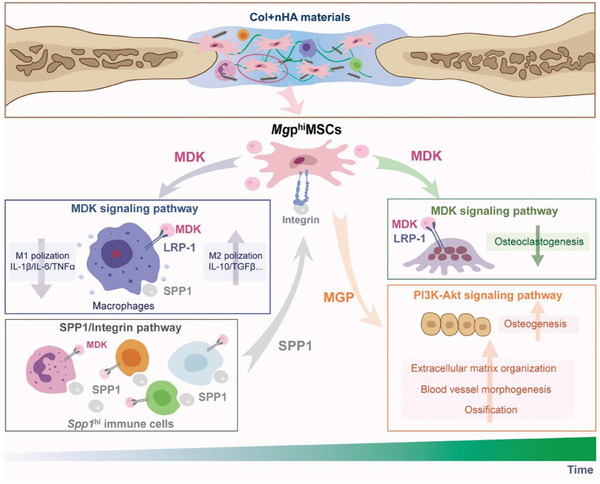
Schematic illustration of the main findings in this study. The figure depicts the cell–cell interactions between *Mgp* ^hi^MSCs and immune cell subsets within the biomaterial‐mediated bone repair process, which is mainly mediated through *Mdk/Lrp1* ligand–receptor pair and SPP1/Integrin pathway. The *Mgp* ^hi^MSCs could enhance osteogenesis via activating the PI3K‐Akt signaling pathway.

The treatment of bone defects suffering from trauma, infections, tumors, or congenital disorders still presents a challenge in orthopedic and craniofacial clinical practice.^[^ ^20^ ^]^ Biomaterials‐based treatment has been proposed as an effective approach to promote bone regeneration. Investigating the spatiotemporal characterization of functional MSCs subpopulation around implanted biomaterials at the early stage of bone regeneration would facilitate the understanding of the ensuring cellular responses and the design of implantable biomaterials. Inspired by that human bone elegantly combines soft organic collagen matrices and hard inorganic nanometer‐sized hydroxyapatite minerals into hierarchical architectures, current studies have incorporated synthetic nHA crystals into collagen matrix to fabricate biomimetic bone repair materials and obtained reliable osteogenic capability in vivo.^[^ ^21^ ^]^ In this study, we applied a Col+nHA hybrid hydrogel to investigate the interactions between biomaterials and autologous cells. The single‐cell atlas of osteoimmune microenvironment within mice calvarial bone defect regions repaired with or without Col+nHA hydrogel composites were constructed via scRNA‐seq after 2 and 7 days of observation. Furthermore, the combined analysis of scRNA‐seq and ST was performed to better reveal the spatial characteristics of functional MSC subpopulations. Our data indicated that *Mgp* ^hi^MSCs were aggregated at the defect regions at the early stage of bone regeneration, exhibiting high osteogenic differentiation potential. The results of cell‐to‐cell communication analysis showed that *Mgp* ^hi^MSCs could modulate the functions of macrophages and osteoclasts, reconstructing the osteoimmune microenvironment via *Mdk/Lrp1* ligand–receptor pair. Meanwhile, the implanted Col+nHA increased the expression levels of *Spp1* in multiple immune cell subsets (macrophages, neutrophils, T cells, NK cells). These *Spp1* ^hi^ immune cells may perform close crosstalk between *Mgp* ^hi^MSCs via SPP1/Integrin signaling pathway (**Figure** **9**). These cellular functions and interactions characterized in this study was expected to serve as the theoretical basis for the design of bone repair materials.

As the driver of bone regeneration, MSCs are identified as a kind of multipotent cells and play essential roles in orchestrating the regeneration process.^[^ ^22^ ^]^ Revealing the underlying repair mechanisms of MSCs during the bone regeneration process and MSCs' response to implanted biomaterials is necessary for the design and optimization of bone repair materials.^[^ ^2a^ ^]^ With the development of lineage‐tracing techniques, several studies have revealed the heterogeneous populations of stem cells involved in bone injury repair.^[^ ^23^ ^]^ An increasing number of studies have revealed that MSCs with different molecular markers, such as *Nestin* ^+^, *LepR* ^+^, and *Msx1* ^+^ MSCs, can migrate to the injured sites and contribute to new bone formation.^[^ ^24^ ^]^ Postnatal *Gli1* ^+^ cells contributed to both normal bone formation and fracture healing.^[^ ^25^ ^]^ *Sox9* marked skeletal progenitors also have been reported to be activated after bone injury and contribute to bone regeneration.^[^ ^26^ ^]^ However, the spatiotemporal characterization of stem cells during the process of bone defect repair and their associated immunomodulatory mechanism are still not clear. Herein, we analyzed the spatial distributions of MSC subclusters and found that the OPs2 subcluster (*Mgp* ^hi^MSCs) exhibited stronger spatial preferences within the bone defect regions compared with other MSC subclusters. The pre‐OBs and pericytes also had a relatively high expression of *Mgp*, while their locations were not specific and their functions were not discussed in this study.

Generally, matrix Gla protein (MGP) was reported as a secreted extracellular protein that belongs to the mineral binding protein family and is associated with bone mineralization.^[^ ^27^ ^]^ Unexpectedly, it was originally identified as a potent physiological suppressor of ectopia calcification in cartilage and vascular tissue.^[^ ^28^ ^]^ On the one hand, MGP inhibits bone morphogenetic protein (BMP) and could maintain the balance between BMP and Notch signaling, promoting a normal brain vasculature. *MGP* deficiency in mice is associated with the medial calcification of arterial walls and cerebral arteriovenous malformations.^[^ ^28c^ ^]^ On the other hand, the biphasic expression pattern of MGP is required for normal chondrocyte differentiation in mammalian growth plate tissue. Interestingly, a high level of MGP may have the same effect as insufficient MGP at different stages of chondrocyte differentiation.^[^ ^29^ ^]^ Substantial MGP upregulation in terminal growth plates chondrocytes may help BMP convert chondrocytes into osteoblasts.^[^ ^28b^ ^]^ What is more, an unexpected role of MGP in osteoclasts has been reported that MGP plays an inhibitory role in osteoclastogenesis and bone resorption.^[^ ^27^ ^]^ Our results demonstrated that the *Mgp* ^hi^MSCs subset could respond to the injury rapidly and migrate into the bone‐defect regions within 48 h. The function analysis showed that *Mgp* ^hi^MSCs performed a series of functions, such as ossification, organization of extracellular matrix, and skeletal system development. Intriguingly, the *Mgp* ^hi^MSCs subset also expresses high levels of *Cxcl12* and *Alp*, which are marker genes of osteo‐CAR cells.^[^ ^12^ ^]^ Our in vitro studies also confirmed that the overexpression of the *MGP* gene in hBMMSCs could promote their osteogenic differentiation potential. These above findings suggested that *Mgp* ^hi^MSCs may play complicated roles in mineralization and bone remodeling at the early stage of bone regeneration.

Another highlight of this study was to characterize the immunomodulatory functions of *Mgp* ^hi^MSCs. Generally, the bone‐defect‐repair process is classically divided into four continuous and overlapping stages, including hemostasis (hours), inflammation (days), repair (1–2 weeks), and remodeling (above 2 weeks).^[^ ^30^ ^]^ When biomaterials were implanted into defect regions, large numbers of immune cells could infiltrate around biomaterials and mediate inflammatory cascades within two days of bone injury.^[^ ^31^ ^]^ Simultaneously, MSCs migrated into the defect areas at the early stage of bone repair and served as the cellular source of the regeneration process.^[^ ^32^ ^]^ Increasing studies also explored the immunomodulatory effects of MSCs, indicating their crucial roles in the mediation of immune cell phenotype polarization.^[^ ^33^ ^]^ The functional MSCs and immune subtypes involved in the process of biomaterials‐medicated bone regeneration and their dynamic response to the implanted biomaterials also remain elusive. In this study, we investigated the heterogeneity of immune cells within the natural and biomaterial‐mediated bone‐defect‐repair microenvironment at 2 and 7 days after injury according to the time frame of immune responses after bone injury. We found that macrophages were more migrated around implanted biomaterials. *Mgp* ^hi^MSCs could evaluate the M2 population of macrophages and mitigate the osteoclastogenesis via *Mdk/Lrp1* ligand–receptor pair. MDK has been reported expressed in a variety of tumor tissue and contributed to tumor‐associated inflammation.^[^ ^34^ ^]^ It serves as an internal modulator of paracrine and autocrine signals and maintains the immune‐resistant state in aggressive tumors.^[^ ^35^ ^]^ The MDK signaling pathway could promote immunosuppressive macrophage differentiation in cancer tissue, while the extent to which MDK regulates the immune‐microenvironment after bone injury is unknown.^[^ ^36^ ^]^ This study demonstrated that *Mdk*/*Lrp1* was enriched at bone defect regions and the expression level of MDK was also upregulated in *Mgp* ^hi^MSCs. Inhibiting the functions of MDK and its receptor, LRP1, resulted in decreased expression of genes associated with M2 polarization (*IL‐10*, *TGF‐β*) and activated osteoclastic differentiation of macrophages. The impact of the *Mdk/Lrp1* ligand‐receptor pair involved in MSC‐macrophage crosstalk provides a potential therapeutic target in immunomodulation of the bone repair process.

It is of note that *Spp1* was ubiquitously upregulated in multiple immune cell subsets around implanted Col+nHA materials compared to the group without materials’ implantation. Previous studies have demonstrated that SPP1 plays a critical role in bone metabolism and homeostasis.^[^ ^37^ ^]^ The expression level of SPP1 on macrophages had a noticeably strong correlation with tumor prognosis.^[^ ^38^ ^]^ The macrophage‐specific knockout of SPP1 could significantly decrease the infiltration of cancer‐associated fibroblasts.^[^ ^39^ ^]^ Here, we highlighted the functional roles of the SPP1/Integrin signaling pathway in cell–cell interactions between multiple immune cells and *Mgp* ^hi^MSCs. These cell–cell interplays provide therapeutic targets for an immune response after the implantation of bone repair materials.

In all, the understanding of cellular and tissue response to implanted biomaterials would promote the development of bone tissue engineering. In previous studies, the heterogeneity, cellular functions, and interactions of immune cells activated by biomaterials have been explored in vivo with the help of scRNA‐seq.^[^ ^40^ ^]^ In contrast to those studies, our study traced the dominant subpopulation of MSCs at the early stage of bone regeneration based on its cellular and spatial characteristics, then revealed its potential osteogenic and immunoregulatory functions, particularly in its interactions with macrophages and osteoclasts. While the network between MSCs and immunocytes around biomaterials is complex and delicate, several immune cells, such as *CD74* ^hi^DC, NK, and T cells, also exhibited a relatively high proportion in the Col+nHA group and performed a close interaction with the OPs2 subset. More efforts should be made to elucidate and verify the dynamic interactions between the above immune cells and MSCs during the process of bone regeneration.^[^ ^41^ ^]^ Armed with this comprehensive understanding of cellular responses to biomaterials, functional subpopulations at the early stage of bone regeneration, as well as cell–cell communications, we can better design biomedical materials to reduce undesirable inflammatory reactions and obtain promoted therapeutic effects.

## 4 Conclusion

In this study, the osteoimmune microenvironment around implanted collagen/nanohydroxyapatite bone repair materials was revealed at a single‐cell level via combined single‐cell and spatial transcriptomics. Specifically, *Mgp* ^hi^MSCs were observed to migrate extensively around implanted biomaterials within the bone defect regions, orchestrating the osteoimmune microenvironment at the early stage of bone regeneration. They could enhance bone regeneration through the PI3K‐Akt signaling pathway, inhibiting the M1 polarization and osteoclastic differentiation of macrophages via the *Mdk/Lrp1* ligand–receptor pair. In turn, *Spp1* ^hi^ immune cells may modulate the function of *Mgp* ^hi^MSCs subsets by activating the SPP1/Integrin signaling pathway. This study clarified the cellular and molecular mechanisms of autologous MSCs' response to the implanted biomaterials at a high resolution, facilitating the innovation of bone tissue engineering.

## 5 Experimental Section

### Material Preparations

To prepare the Col+nHA hydrogel composites, nHA nanoparticles (Sigma–Aldrich, USA) were suspended in deionized water at a concentration of 50 mg mL^−1^ and sonicated for 30 min. Then freeze‐dried collagen (Hebei KaoLiSen Biotechnology Co., Ltd., China) was dissolved into the above nanoparticles suspension at a concentration of 50 mg mL^−1^ under simultaneous stirring to obtain a homogeneous hydrogel composite. The sterilized collagen or nHA particles were respectively suspended in phosphate buffered saline (PBS) solution at a concentration of 50 mg mL^−1^ and used as the control groups (Col and nHA).

### Animal Studies

C57BL/6J mice (6‐weeks‐old, male, 18–20g) were used to establish calvarial defect models. All animal experiments were approved by the Peking University Animal Care and Use Committee (Project Number: LA2022040) and performed in accordance with the institutional animal guidelines. Briefly, after general anesthesia administration and disinfection, a sagittal incision was made to expose the calvarium of mice. The periosteum covering the bone was removed and two circular full‐thickness bone defects (diameter: 3 mm) were created on the bilateral parietal bone of mice by using a trephine bur under low‐speed drilling and copious saline irrigation. The bone debris was removed carefully without damage to the underlying dura mater. The Col hydrogel, nHA solution, and Col+nHA hydrogel composites were injected into the defect regions respectively. The excess materials outside the defect regions were removed with gauze in the nHA group. Finally, the incision sites were sutured with 6–0 nylon sutures. After recovering for 2 and 7 days, calvarial bones including hydrogel composites were together harvested for scRNA‐seq. 7 days' samples were also used for ST analysis. 12 weeks after surgery, the mice were euthanatized and then calvarial bones were harvested and fixed in 4% paraformaldehyde.

Micro‐computerized tomography (micro‐CT) scanning was obtained using the Inveon micro‐CT system (Siemens, MUC, Germany). Complete skulls were scanned at energy of 60 kVp, intensity of 220 µA, and effective pixel size of 9.21 µm. The bone mineral density (BMD), bone volume/tissue volume (BV/TV), and trabecular number (Tb.N.) were quantified via a multimodal 3D visualization software (Inveon Research Workplace, Siemens, MUC, Germany). Thereafter, the decalcified samples were embedded in paraffin and cut into 5 µm thick sections for histological evaluation. Hematoxylin and eosin (HE), Masson's trichrome (Masson), and Safranin O‐fast green staining were performed to detect new bone formation. The Masson staining kit (Cat#1006, Servicebio, China) and the Safranin O‐fast green staining kit (Cat#G1053, Servicebio, China) were used for staining according to the manufacturer's protocols. For Masson staining, the slides were stained with hematoxylin, with Ponceau S acid fuchsin, and aniline blue in order. The differential processes were performed using an acid aqueous solution after each time staining to control the degree of coloration.

The calvarial bones in the Col+nHA group were harvested after 7 days of observation for immunohistochemical staining. The samples were decalcified, embedded in paraffin, and cut into 5 µm thick sections. Antigen retrieval was performed in 0.01 m sodium citrate buffer (pH 6.0), heating in a water bath at 95 °C for 30 min. The MGP monoclonal antibody (Cat#60055‐1‐Ig, Proteintech, USA) was used as the primary antibody, and the biotinylated anti‐mouse IgG (Cat#G1214, Servicebio, China) was used as the secondary antibody. Then the slides were visualized using diaminobenzidine and the nuclei were counterstained with hematoxylin for observation.

### Single‐Cell RNA Sequencing

The mice bone‐defect samples were obtained by cutting off the calvarial bone defect areas together with hydrogel composites (circular; diameter, 3 mm). Soft tissue was gently removed and a total of 12 (six mice) were harvested in each group. The harvested samples were cut into small pieces and digested with prewarmed PBS containing 4% FBS (Gibco), 1% penicillin–streptomycin (P/S) (v/v) (Gibco), 1.0 mg/mL type II collagenase, and 0.5 mg mL^−1^ type IV collagenase (Worthington, Lakewood, NJ, USA) at 37 °C for 1 h (300 rpm). Then the samples were collected into centrifuge tubes through the 40‐µm filters, and the supernatant was removed after centrifugation. Erythrocytes were removed by incubating with RBC lysis buffer (Solarbio, China) on ice for 15 min. The remaining cells were then washed and incubated with 7AAD viability staining solution (Biolegend, San Diego, CA, USA) for 10 min. After cell sorting, the dead cells and debris were removed, and the obtained single‐cell suspension was used for scRNA‐seq. Cell viability was assessed with Trypan blue exclusion on a TC20 automated cell counter (BIO‐RAD, Hercules, CA, USA) and showed a >80% viability. Droplet‐based single‐cell RNA‐seq sample libraries were prepared with the 10× Genomics Chromium Single Cell 3′v3.1 according to the manufacturer's instructions and sequenced using the Illumina HiSeq 6000 platform (Novogene, Tianjin, China).

### scRNA‐Seq Data Processing

For 10× dataset, 10× Genomics Cell Ranger pipeline (v4.0.0; https://www.10xgenomics.com/) with default mapping parameters to aggregate raw data, filter low‐quality reads, align reads to the mouse reference genome (mm10), assign cell barcodes, and count unique molecular identifiers (UMIs) was used. Then the expression matrix was imported into the Seurat package (v3.1; http://satijalab.org/seurat/). A gene with expression in more than three cells was considered as expressed. Cells with fewer than 200 detected genes and high mitochondrial gene‐expression fractions were filtered out. Seurat alignment method canonical correlation analysis (CCA) was utilized for integrated analysis.^[^ ^42^ ^]^ Highly variable genes (HVGs) were selected via the FindVariableGenes function in the Seurat package. Based on the HVGs detected, dimension reduction was conducted and visualized by uniform manifold approximation and projection (UMAP) or t‐distributed stochastic neighbor embedding (t‐SNE).

Marker genes for each cluster were identified using the FindAllMarkers function in Seurat and only genes with adjusted *p*‐value < 0.05 (determined by the Wilcoxon rank‐sum test) were considered. Subsequently, GO and KEGG enrichment analyses of identified marker genes were implemented by the clusterProfiler package (v3.4.4). GO and KEGG terms with corrected *p*‐value < 0.05 were considered significantly enriched. Cell types of different subclusters were annotated based on their marker genes and associated GO items. To obtain the high‐resolution map of MSCs and macrophages, the re‐clustering of major clusters was performed according to the workflow as above. To reveal the differentiation trajectory of MSCs and macrophages, the pseudotime analysis was performed in Monocle2. The RNA velocity of MSC subsets was performed via scVelo. CellChat, a tool enabled to quantitatively infer and analyze intercellular communication networks, was used for receptor–ligand analysis.

### Spatial Transcriptomics

After 7 days' observation, the calvarial bone‐defect samples were rapidly harvested, rinsed with pre‐cooled PBS solution, and frozen in OCT (Sakura, USA). Serial cryosections were cut from the OCT‐embedded samples for HE staining and histopathological assessment. Total RNA of 100–200 µm thick sections was extracted using the RNeasy Mini Kit (Qiagen, USA) for quality control. The RNA integrity number (RIN) was determined using an Agilent 2100 Bioanalyzer (USA). Only samples with RIN ≥ 7 were qualified for the transcriptomic study. Then permeabilization time was optimized according to the Visium Spatial Tissue Optimization User Guide (CG000238, 10X Genomics). Spatial transcriptomics was conducted using 10× Visium Spatial Gene Expression Slide & Reagent Kit according to the manufacturer's protocols (CG000239, 10× Genomics). The libraries were sequenced on the Illumina Novaseq 6000 platform (Novogene, Tianjin, China) at an average depth of 300 million read‐pairs per sample. Genome alignment was conducted using the 10× Genomics SpaceRanger pipeline (v1.2.0) and the reads were aligned to the pre‐built mouse reference genome mm10. The filtered gene‐spots matrix and the fiducial‐aligned low‐resolution image were used for down‐streaming data analyses (including gene expression normalization, dimensionality reduction, spot clustering, and differential expression analysis) using Seurat package (v3.1) in R. The clusterProfiler R package was used to calculate enrichment tests for candidate gene sets based on the hypergeometric distribution. Then the combined analysis of scRNA‐seq and ST datasets was performed in Seurat (v4.0) with the FindTransferAnchors and TransferData functions. Spatial morphological gene expression (SME) normalization was applied to improve individual spot expression quality in stLearn (v0.3.1). Ligand–receptor analysis and visualizations were performed using the stLearn CCI pipeline.

### Cell Culture

The primary hBMMSCs were obtained from ScienCell Company (San Diego, CA, USA). Proliferation medium (PM) containing Minimum Essential Medium α (α‐MEM, Gibco, Grand Island, NY, USA), 10% (v/v) FBS, and 1% (v/v) penicillin/streptomycin was used for cell culture. The osteogenic medium (OM) consisting of 10 mm β‐glycerophosphate (Sigma–Aldrich, USA), 50 µg mL^−1^ ascorbate acid (Sigma–Aldrich, USA), 10 nm dexamethasone (Sigma–Aldrich, USA) was used for hBMMSCs osteogenic differentiation and refreshed every 3 days.

The human monocyte cell line THP‐1 was obtained from the American Type Culture Collection (ATCC, VA, USA), and cultured with RPMI 1640 (Gibco, USA) supplemented with 10% (v/v) FBS and 1% (v/v) penicillin/streptomycin at 37 °C under 5% humidified CO_2_. Macrophages were differentiated from THP‐1 cells using serum‐free Dulbecco' smodified Eagle medium (DMEM, Gibco, USA) supplemented with 10 ng mL^−1^ phorbol 12‐myristate 13‐acetate (PMA, Sigma‐Aldrich, USA). After 48 h, the THP‐1‐derived macrophages were further cultured with condition medium (CM) of hBMMSCs or co‐cultured with hBMMSCs. For osteoclastic differentiation of THP‐1‐derived macrophages, 50 ng mL^−1^ RANKL (Cat#300‐25‐1MG, PeproTech, USA), and 30 ng mL^−1^ M‐CSF (Cat#310‐01‐1MG, PeproTech, USA) were further supplemented in the medium for a 7 or 14‐days' osteoclastic induction and replaced every 2 days.

The Transwell coculture assays were performed using 12‐well Transwell inserts (0.4 µm, Corning Costar, Tewksbury, USA). THP‐1 cells were seeded into the bottom of each well and supplemented with PMA for induction. After 48 h, hBMMSCs were seeded into the Transwell inserts. To investigate the immunoregulatory of MDK, DMSO, and iMDK (500 nm, Cat#T9460, TargetMol, USA) were added into the co‐culture system.

### RNA Interference and Lentiviral Transfection

Small‐interfering RNAs (siRNA) targeting *MGP*, *LRP1*, and their negative control (si‐NC), *MGP* plasmid, and vector were purchased from GenePharma (Suzhou, China). Lipofectamine 3000 transfection kit (Invitrogen) was used for transfection. *MGP*‐overexpressing lentivirus (MGP) and vector were purchased from GenePharma (Suzhou, China). Viral supernatants at a MOI of 100 with 5 mg mL^−1^ polybrene were added into the cell culture for transfection. Puromycin (1 µg mL^−1^, Cat#16561‐29‐8, Sigma–Aldrich, USA) was applied to select stably transfected cells after transfection 48–72 h. The transfection efficiency was verified via qRT‐PCR and western blots.

### Drugs Treatment

10 µm PI3K/AKT‐IN‐1 (Cat#HY‐144806, MedChemExpress, China) and 7 µm miltefosine (Cat#HY‐13685, MedChemExpress, China) was used to inhibit PI3K‐Akt signaling pathway.

### Transwell Assay

The 24‐well Transwell inserts (8 µm, Corning Costar, Tewksbury, USA) were used for the assay. The hBMMSCs transfected with vector, hBMMSCs transfected with *MGP*‐overexpressing lentivirus (MGP), as well as hBMMSCs transfected with *MGP*‐overexpressing lentivirus and then interfered with si*MGP*‐1 (MGP+siMGP) were resuspended in 100 µL of serum‐free α‐MEM and added into the upper chamber (2 × 10^4^ per well). 100 µL collagen/nHA hydrogel composites and 500 µL of α‐MEM (2% FBS) were added into the lower chamber. The group cultured without hydrogel composites was used as the blank group. The recombinant human osteopontin/SPP1 protein (Cat#RP00989, Abclonal, China) was applied to investigate the chemotaxis effects of SPP1. After being cultured within 24 h, the cotton swab was used to wipe off the hBMMSCs on the upper surface of the chamber. Then inserts were rinsed with PBS, fixed with 95% cold ethanol for 30 min, and stained with 0.1% crystal violet solution. The migrated cells were observed in ten random views and counted using Image J software.

### Alkaline Phosphatase, Alizarin Red S, and TRAP staining

ALP staining of hBMMSCs was performed according to the protocols of the NBT/BCIP staining kit (Beytime, China). After 14 days of osteogenic induction, 1% Alizarin red (Sigma–Aldrich, USA) buffer was used for staining. To quantify mineral accumulation, a 100 mm cetylpyridine (Sigma–Aldrich, USA) solution was added to dissolve the cells and an absorbance of 490 nm was used for quantification. THP‐1 cells were fixed in 3.7% paraformaldehyde for 10 min, treated with ice ethanol/acetone solution for 1 min, and stained using a TRAP staining kit (FUJIFILM Wako, Japan).

### Quantitative Reverse Transcription Polymerase Chain Reaction (qRT‐PCR)

Total RNA was extracted from the culture cells using TRIzol Reagent (Invitrogen, Carlsbad, CA, USA). A NanoDrop 8000 spectrophotometer (Thermo Fisher Scientific) was used to determine the purity and concentration of total RNA. In brief, total RNA was reverse transcribed into cDNA using the PrimeScript RT kit (AG Scientific, China), and then the cDNA was used to perform qRT‐PCR using SYBR Green Master Mix (YEASEN, China). The primer sequences of genes are shown in Table S1 (Supporting Information). GAPDH was regarded as the internal reference. The level of gene expression was analyzed using the 2^−∆∆Ct^ method.

### Western Blot

To detect the expression levels of protein, cells were lysed in a lysis buffer containing 1% proteinase and phosphatase inhibitor (NCM, China). Protein extracts were subjected to 10% SDS‐PASE and transferred to a polyvinylidene fluoride membrane. The membrane was incubated with the primary antibodies overnight, then was incubated with peroxidase‐conjugated secondary antibodies for 1h at room temperature. The visualized immunoreactive protein bands were detected using an enhanced chemiluminescence (ECL) kit (NCM, China).

### Immunofluorescent Staining

The samples were fixed in 4% paraformaldehyde for 30 min, permeabilized with 0.25% Triton‐X for 7 min, and blocked by bovine serum albumin (5% w/v) for 1 h. Subsequently, cells were incubated with primary antibodies against iNOS (Cat#340668, zen‐bio, China), ARG‐1 (Cat#A4923, Abclonal, China), TRAP (Cat#A2748, Abclonal, China), c‐Fos (Cat#A24620, Abclonal, China), LRP1 (Cat#A1439, Abclonal, China) overnight, incubated with Rhodamine (TRITC)‐conjugated secondary antibodies (Cat# SA00007‐2, Proteintech, USA) or CoraLite488‐conjugated secondary antibodies (Cat#SA00013‐2, Proteintech, USA) for 1 h, then stained with Phalloidin‐iFluor 594 reagent (Cat#AB176757, abcam, USA). After washing with PBS, the cells were mounted with mounting media containing DAPI and viewed by a fluorescence microscope (Olympus, Tokyo, Japan) or LSM710 confocal laser scanning microscope (Zeiss, Oberkochen, Germany).

### Bulk RNA Sequencing and Analysis

After 4 days of co‐culture, the total RNA of THP‐1 cells co‐cultured with hBMMSCs was extracted using TRIzol reagent (Invitrogen, Carlsbad, CA, USA). RNA sequencing and data processing were performed by Novogene Co. Ltd. (Tianjin, China) according to the protocol described earlier. DEGs were identified when the *p‐*value was <0.05 and the fold change was > 2. GO term enrichment and KEGG enrichment analysis displayed significantly enriched biological processes and pathways when the Q value was less than 0.05.

### Quantification and Statistical Analysis

Results were recorded as a mean ± standard error of the mean (SD). Statistical analysis was performed using GraphPad Prism software version 9.0. Statistically significant differences were assessed by unpaired two‐tailed Student's *t* test (between two groups) and one‐way analysis of variance (ANOVA) with Turkey's post hoc test (among three groups). Statistical significance was defined as *p < 0.05, **p < 0.01.

## Conflict of Interest

The authors declare no conflict of interest.

## Author Contributions

Z.W. conceptualized the study, carried out the laboratory work, collected data carried out the analysis, and wrote the manuscript; X.B., X.W., and X.G. carried out the laboratory work and collected data; X.W., M.Z., and Y.F. performed functional experiments; Y.L., P.Z., X.Z., R.Y., and Y.L. conducted the study, provided the provision of study material; L.L., Y.Z. conceptualized the study, and took up the responsibility for the integrity of the data analysis, manuscript writing, financial support, and final approval of manuscript. All authors have read and approved the article.

## Supporting information

Supporting Information

## Data Availability

The data that support the findings of this study are available from the corresponding author upon reasonable request.
