# Supplementary material for: Mgp High‐Expressing MSCs Orchestrate the Osteoimmune Microenvironment of Collagen/Nanohydroxyapatite‐Mediated Bone Regeneration
Source: Adv Sci (Weinh). 2024 Apr 8;11(23):2308986. doi: 10.1002/advs.202308986 (PMC11187922; doi:10.1002/advs.202308986)
Supplement: Supplementary file 1 — Supporting Information [file ADVS-11-2308986-s001.pdf]

ADVANCED SCIENCE
Open Access

# Supporting Information

for *Adv. Sci.*, DOI 10.1002/advs.202308986

*Mgp* High-Expressing MSCs Orchestrate the Osteoimmune Microenvironment of Collagen/Nanohydroxyapatite-
Mediated Bone Regeneration

*Zhuqing Wan, Xiaoqiang Bai, Xin Wang, Xiaodong Guo, Xu Wang, Mo Zhai, Yang Fu, Yunsong Liu, Ping Zhang, Xiao Zhang, Ruili Yang, Yan Liu, Longwei Lv* and Yongsheng Zhou**

# Supplementary Materials for

**_Mgp_ high-expressing MSCs orchestrate the osteoimmune microenvironment of collagen/nanohydroxyapatite-mediated bone regeneration**

Zhuqing Wan[1,3], Xiaoqiang Bai[1,3], Xin Wang[1,3], Xiaodong Guo[1,3], Xu Wang[1,3], Mo Zhai[1,3], Yang Fu[1,3], Yunsong Liu[1,3], Ping Zhang[1,3], Xiao Zhang[1,3], Ruili Yang[2,3], Yan Liu[2,3], Longwei Lv[*,1,3], Yongsheng Zhou[**,1,3]

Correspondence to: lvlw@bjmu.edu.cn (Longwei Lv); kqzhouysh@hsc.pku.edu.cn (Yongsheng Zhou)

[1]Department of Prosthodontics, Peking University School and Hospital of Stomatology, Haidian District, Beijing 100081, China.

[2]Department of Orthodontics, Peking University School and Hospital of Stomatology, Haidian District, Beijing 100081, China.

[3]National Center for Stomatology, National Clinical Research Center for Oral Disease, National Engineering Research Center of Oral Biomaterials and Digital Medical Devices, Beijing Key Laboratory of Digital Stomatology, NHC Key Laboratory of Digital Stomatology, NMPA Key Laboratory of Digital Stomatology, Chinese Academy of Medical Sciences, Haidian District, Beijing 100081, China.

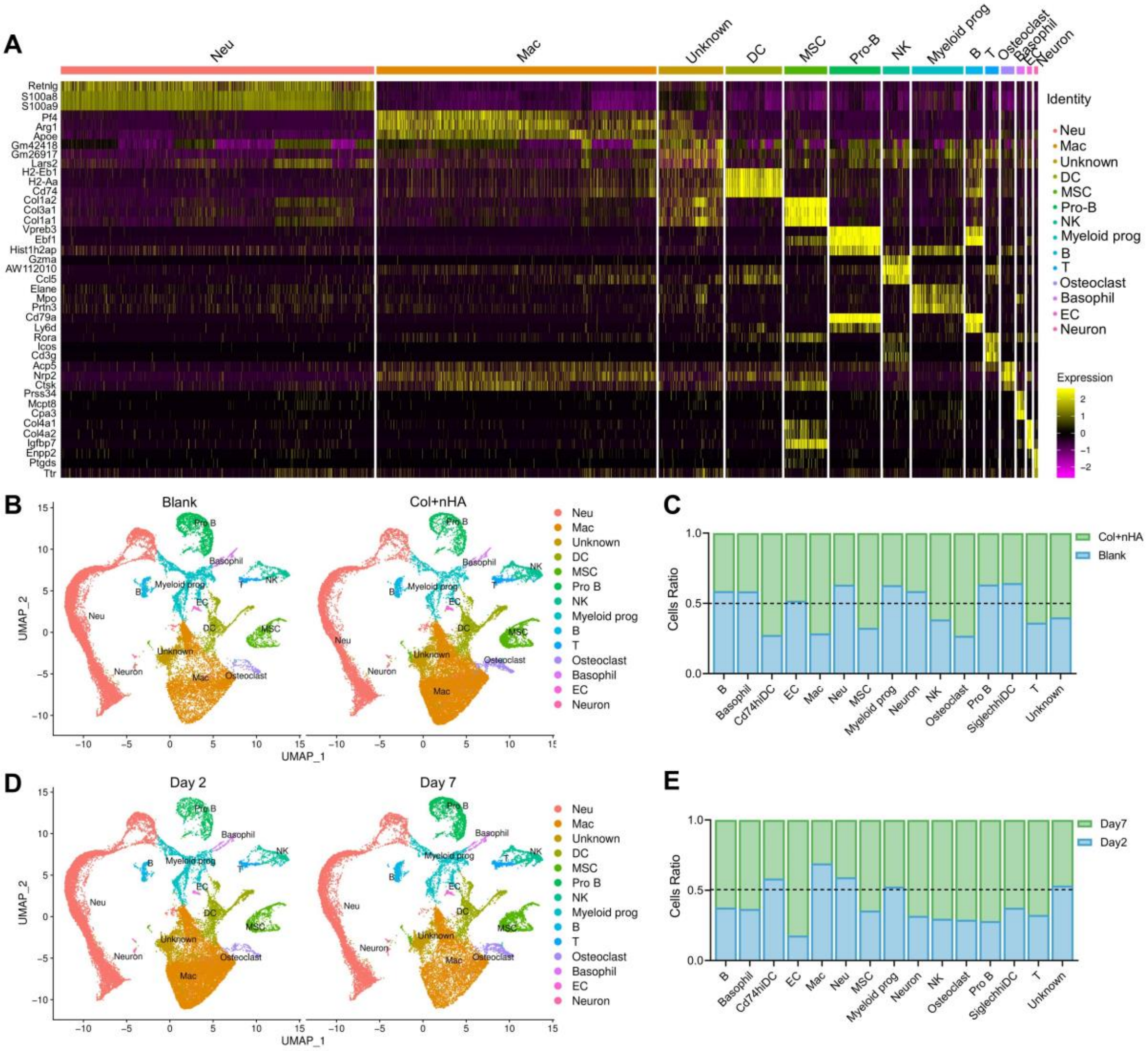


**Figure S1. Characterization of each cluster in mice calvarial bone defects repaired with or without bioactive Col+nHA hydrogel composites.** (A) Heatmap showing the differentially expressed genes (DEGs) of different cell types. The UMAP plots (B) and cell ratios (C) revealing the comparison of 14 clusters between Blank and Col+nHA samples. The UMAP plots (D) and cell ratios (E) revealing the comparison of 14 clusters between day 2 and day 7 samples.

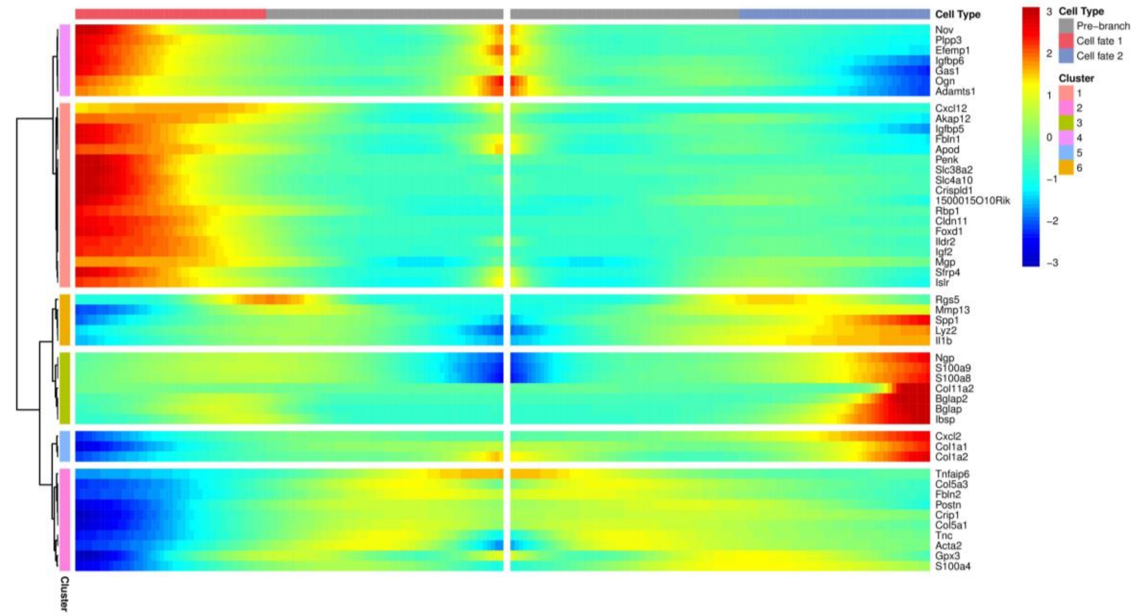


**Figure S2.** Clustered heatmap of differential genes at two trajectory branch points ($P$＜1.0-8e).

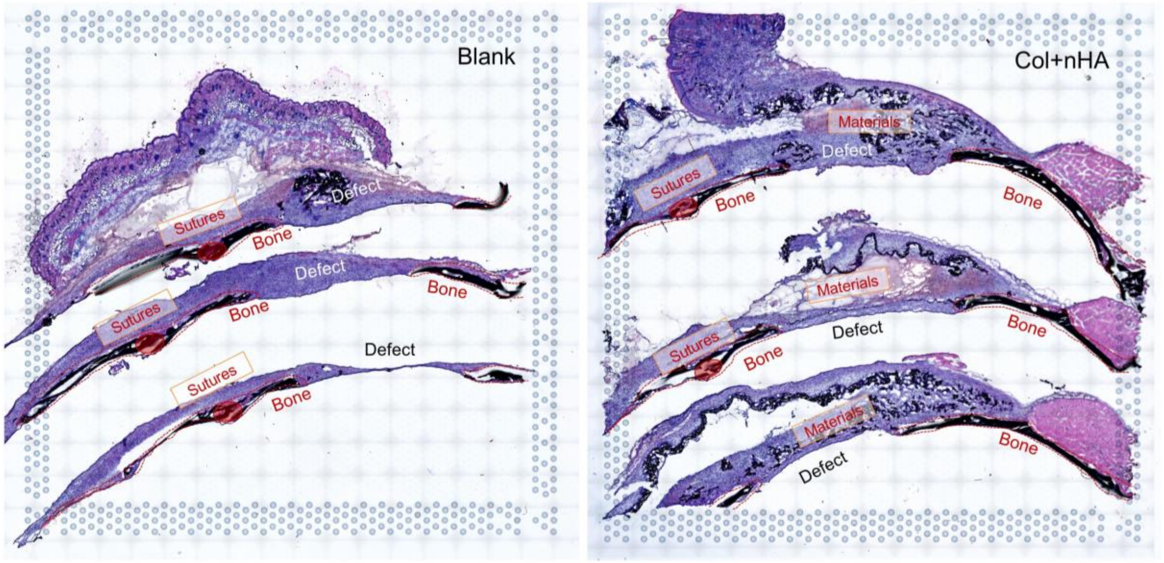


**Figure S3.** HE staining of the cryosection slices of calvarial bone defects repaired with or without bioactive Col+nHA hydrogel composites. The red circles marked the suture structures.

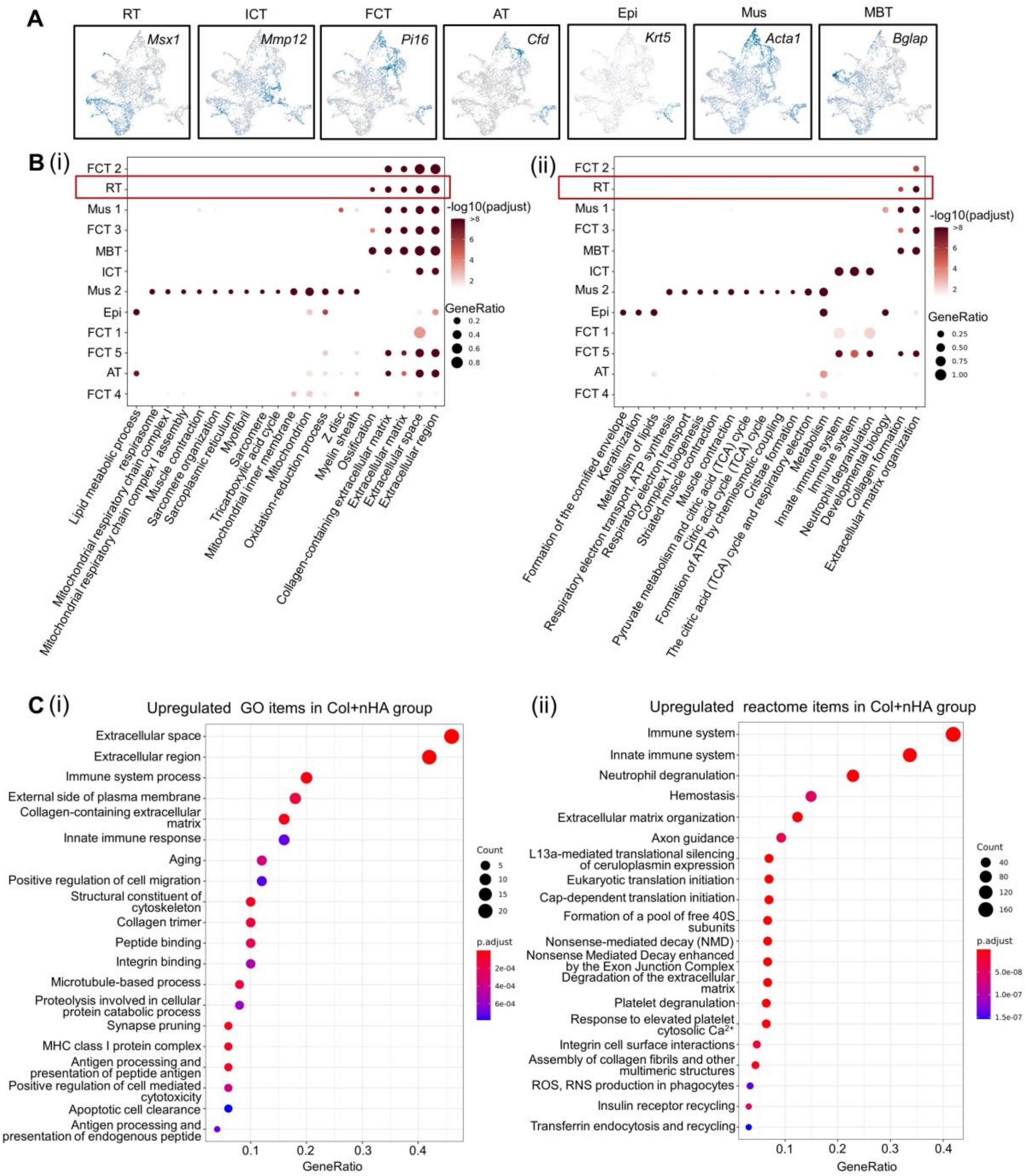


**Figure S4.** (A) UMAPs color-coded for expression of key cell-type markers to define the spot types in ST dataset. FCT: fibrous connective tissue. RT: regenerative tissue. Mus: muscle. MBT: mature bone tissue. ICT: inflammatory connective tissue. Epi: epidermis. AT: adipose tissue. (B) Enriched Gene ontology (GO) terms (i) and Reactome terms (ii) of different spots types. (C) Upregulated GO and reactome items Col+nHA group of RT cluster.

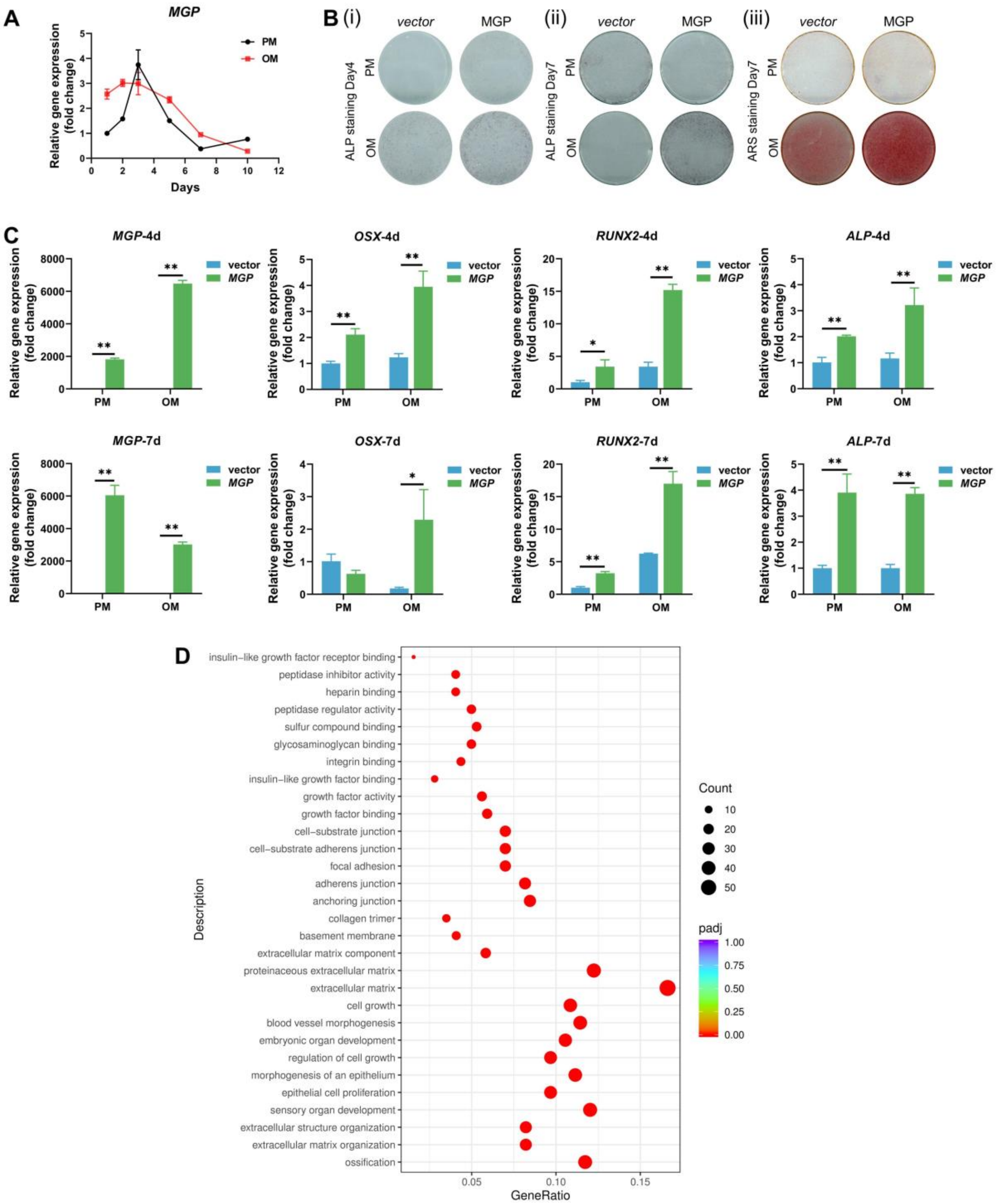


**Figure S5. MGP overexpression enhances osteogenic differentiation of hBMMSCs *in vitro*.** (A) MGP expression during the osteogenic differentiation of hBMMSCs determined by RT-qPCR. (B) ALP and ARS staining of MGP overexpressing hBMMSCs constructed via mutant plasmids at different time points. (C) MGP overexpression promoted the mRNA expression of osteogenic

genes. (D) Upregulated GO items of OPs2 subcluster. PM: proliferation medium; OM: osteogenic medium.

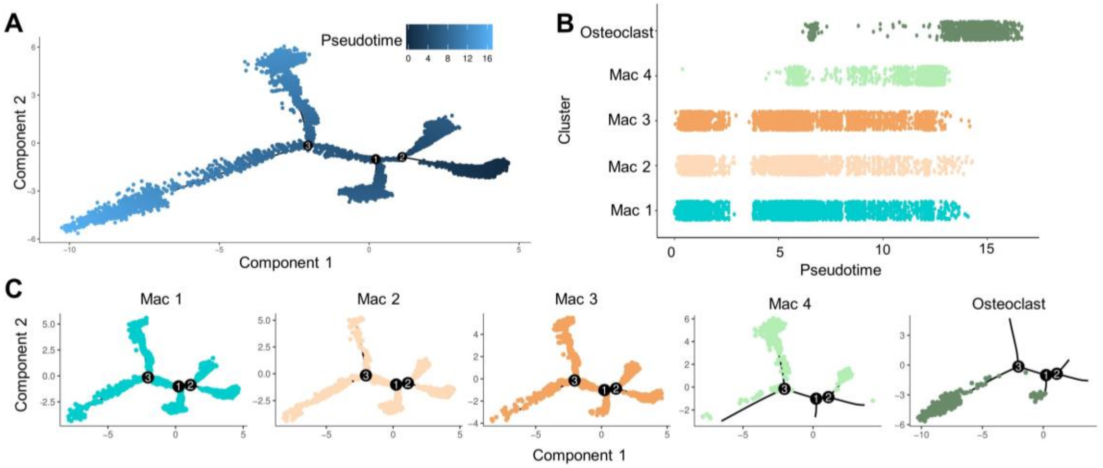


**Figure S6. The multilineage differentiation capacity of macrophages.** (A) The differentiated direction of macrophage subclusters along the pseudotime. (B) Distribution of each macrophage subcluster along the pseudotime. (C) Pseudotime lineage trajectory analysis demonstrating the relationships of macrophage subclusters, color coded by subclusters.

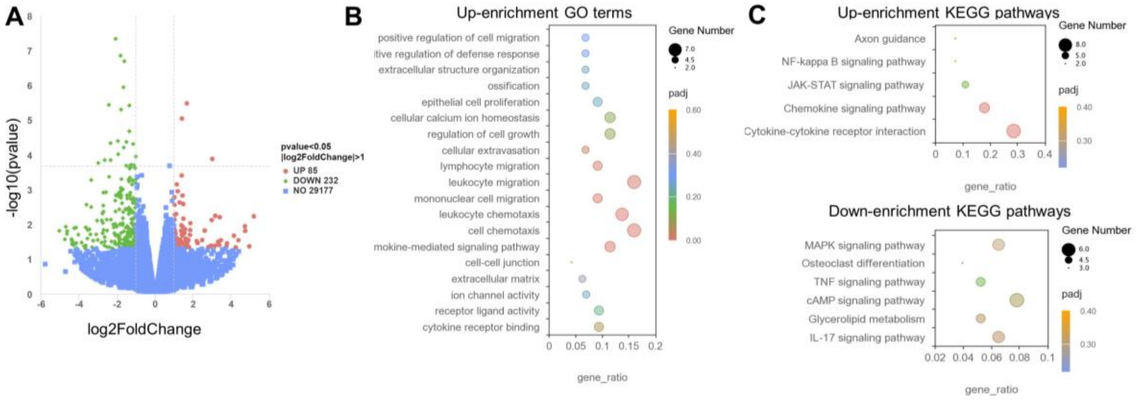


**Figure S7. RNA-seq analysis of THP-1-derived macrophages cultured with the conditional medium (CM) of MGP overexpressing hBMMSCs.** (A) Volcano plot showing the up and down-regulated genes in THP-1 cells. (B) Up-regulated GO items in THP-1 cells cultured with MGP overexpressed hBMMSCs-derived CM. (C) Up and down-regulated KEGG items in THP-1 cells cultured with MGP overexpressed hBMMSCs-derived CM.

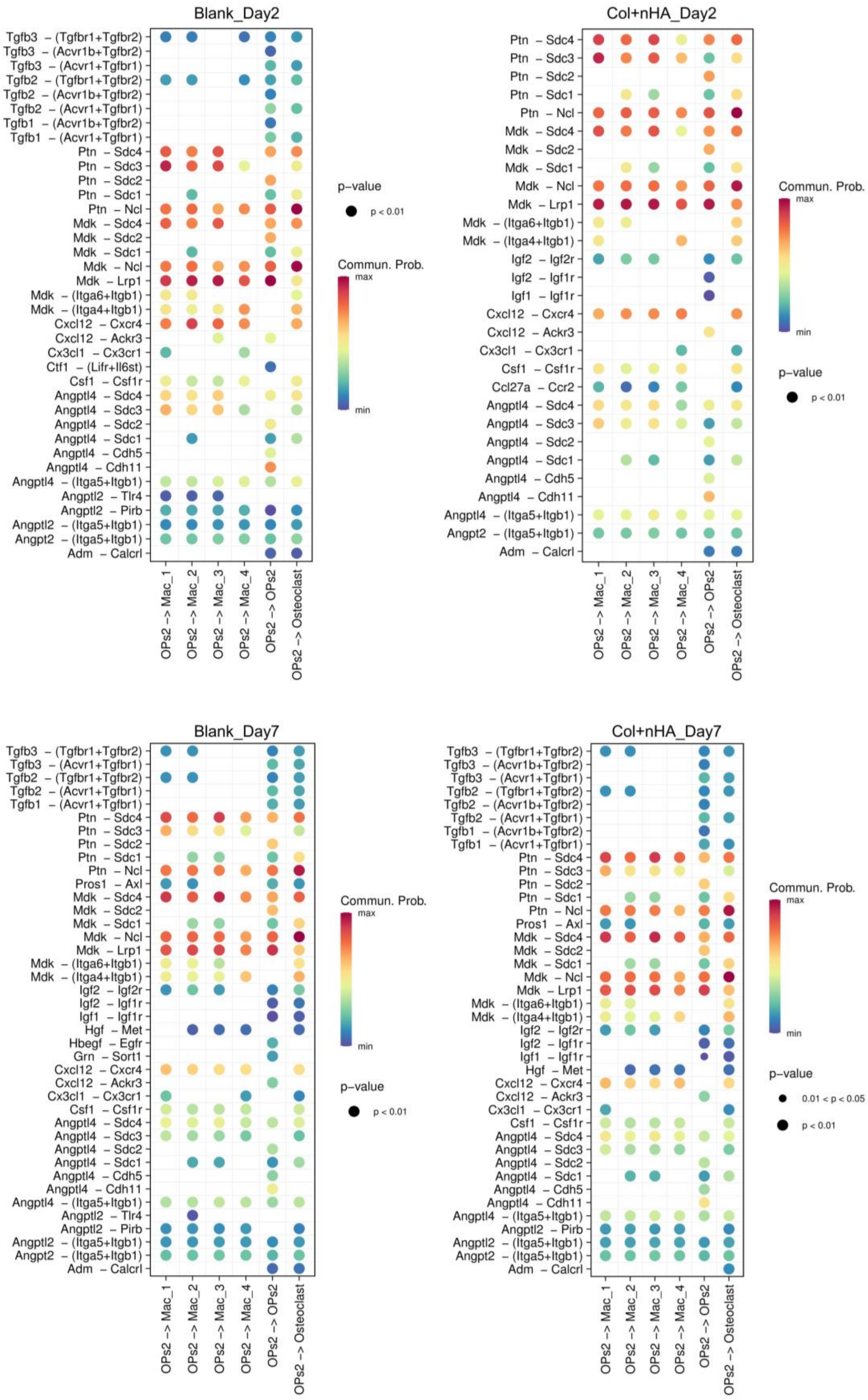
Blank_Day2
Tgfb3 – (Tgfbr1+Tgfbr2)
Tgfb3 – (Acvr1b+Tgfbr2)
Tgfb3 – (Acvr1+Tgfbr1)
Tgfb2 – (Tgfbr1+Tgfbr2)
Tgfb2 – (Acvr1b+Tgfbr2)
Tgfb2 – (Acvr1+Tgfbr1)
Tgfb1 – (Acvr1b+Tgfbr2)
Tgfb1 – (Acvr1+Tgfbr1)
Ptn – Sdc4
Ptn – Sdc3
Ptn – Sdc2
Ptn – Sdc1
Ptn – Ncl
Mdk – Sdc4
Mdk – Sdc2
Mdk – Sdc1
Mdk – Ncl
Mdk – Lrp1
Mdk – (Itga6+Itgb1)
Mdk – (Itga4+Itgb1)
Cxcl12 – Cxcr4
Cxcl12 – Ackr3
Cx3cl1 – Cx3cr1
Ctf1 – (Lifr+Il6st)
Csf1 – Csf1r
Angptl4 – Sdc4
Angptl4 – Sdc3
Angptl4 – Sdc2
Angptl4 – Sdc1
Angptl4 – Cdh5
Angptl4 – Cdh11
Angptl4 – (Itga5+Itgb1)
Angptl2 – Tlr4
Angptl2 – Pirb
Angptl2 – (Itga5+Itgb1)
Angpt2 – (Itga5+Itgb1)
Adm – Calcrl
OPs2 -> Mac_1
OPs2 -> Mac_2
OPs2 -> Mac_3
OPs2 -> Mac_4
OPs2 -> OPs2
OPs2 -> Osteoclast
p-value
p < 0.01
Commun. Prob.
max
min
Col+nHA_Day2
Ptn – Sdc4
Ptn – Sdc3
Ptn – Sdc2
Ptn – Sdc1
Ptn – Ncl
Mdk – Sdc4
Mdk – Sdc2
Mdk – Sdc1
Mdk – Ncl
Mdk – Lrp1
Mdk – (Itga6+Itgb1)
Mdk – (Itga4+Itgb1)
Igf2 – Igf2r
Igf2 – Igf1r
Igf1 – Igf1r
Cxcl12 – Cxcr4
Cxcl12 – Ackr3
Cx3cl1 – Cx3cr1
Csf1 – Csf1r
Ccl27a – Ccr2
Angptl4 – Sdc4
Angptl4 – Sdc3
Angptl4 – Sdc2
Angptl4 – Sdc1
Angptl4 – Cdh5
Angptl4 – Cdh11
Angptl4 – (Itga5+Itgb1)
Angpt2 – (Itga5+Itgb1)
Adm – Calcrl
OPs2 -> Mac_1
OPs2 -> Mac_2
OPs2 -> Mac_3
OPs2 -> Mac_4
OPs2 -> OPs2
OPs2 -> Osteoclast
Commun. Prob.
max
min
p-value
p < 0.01
Blank_Day7
Tgfb3 – (Tgfbr1+Tgfbr2)
Tgfb3 – (Acvr1+Tgfbr1)
Tgfb2 – (Tgfbr1+Tgfbr2)
Tgfb2 – (Acvr1+Tgfbr1)
Tgfb1 – (Acvr1+Tgfbr1)
Ptn – Sdc4
Ptn – Sdc3
Ptn – Sdc2
Ptn – Sdc1
Ptn – Ncl
Pros1 – Axl
Mdk – Sdc4
Mdk – Sdc2
Mdk – Sdc1
Mdk – Ncl
Mdk – Lrp1
Mdk – (Itga6+Itgb1)
Mdk – (Itga4+Itgb1)
Igf2 – Igf2r
Igf2 – Igf1r
Igf1 – Igf1r
Hgf – Met
Hbegf – Egfr
Grn – Sort1
Cxcl12 – Cxcr4
Cxcl12 – Ackr3
Cx3cl1 – Cx3cr1
Csf1 – Csf1r
Angptl4 – Sdc4
Angptl4 – Sdc3
Angptl4 – Sdc2
Angptl4 – Sdc1
Angptl4 – Cdh5
Angptl4 – Cdh11
Angptl4 – (Itga5+Itgb1)
Angptl2 – Tlr4
Angptl2 – Pirb
Angptl2 – (Itga5+Itgb1)
Angpt2 – (Itga5+Itgb1)
Adm – Calcrl
OPs2 -> Mac_1
OPs2 -> Mac_2
OPs2 -> Mac_3
OPs2 -> Mac_4
OPs2 -> OPs2
OPs2 -> Osteoclast
Commun. Prob.
max
min
p-value
p < 0.01
Col+nHA_Day7
Tgfb3 – (Tgfbr1+Tgfbr2)
Tgfb3 – (Acvr1b+Tgfbr2)
Tgfb3 – (Acvr1+Tgfbr1)
Tgfb2 – (Tgfbr1+Tgfbr2)
Tgfb2 – (Acvr1b+Tgfbr2)
Tgfb2 – (Acvr1+Tgfbr1)
Tgfb1 – (Acvr1b+Tgfbr2)
Tgfb1 – (Acvr1+Tgfbr1)
Ptn – Sdc4
Ptn – Sdc3
Ptn – Sdc2
Ptn – Sdc1
Ptn – Ncl
Pros1 – Axl
Mdk – Sdc4
Mdk – Sdc2
Mdk – Sdc1
Mdk – Ncl
Mdk – Lrp1
Mdk – (Itga6+Itgb1)
Mdk – (Itga4+Itgb1)
Igf2 – Igf2r
Igf2 – Igf1r
Igf1 – Igf1r
Hgf – Met
Cxcl12 – Cxcr4
Cxcl12 – Ackr3
Cx3cl1 – Cx3cr1
Csf1 – Csf1r
Angptl4 – Sdc4
Angptl4 – Sdc3
Angptl4 – Sdc2
Angptl4 – Sdc1
Angptl4 – Cdh5
Angptl4 – Cdh11
Angptl4 – (Itga5+Itgb1)
Angptl2 – Pirb
Angptl2 – (Itga5+Itgb1)
Angpt2 – (Itga5+Itgb1)
Adm – Calcrl
OPs2 -> Mac_1
OPs2 -> Mac_2
OPs2 -> Mac_3
OPs2 -> Mac_4
OPs2 -> OPs2
OPs2 -> Osteoclast
Commun. Prob.
max
min
p-value
0.01 < p < 0.05
p < 0.01

**Figure S8.** Unique ligand-receptor pairs between OPs2 and macrophage/osteoclast clusters in two groups at different timepoints.

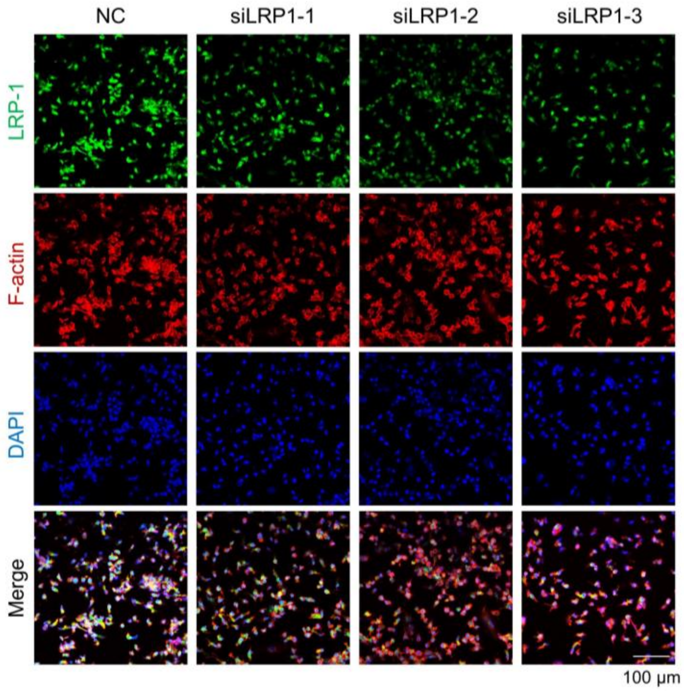


**Figure S9.** The expression level of LRP1 receptor in THP-1 derived macrophages transfected with small-interfering RNAs (siRNA) targeting LRP1 observed via laser-scanning confocal microscope.

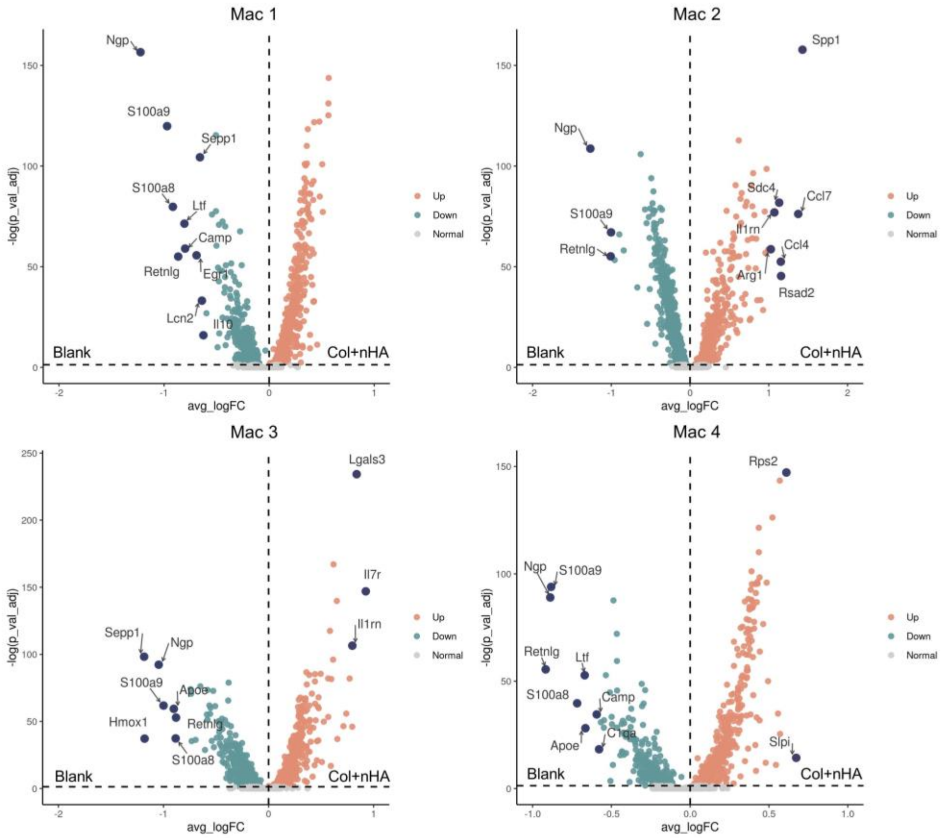


**Figure S10.** Volcano plots showing DEGs of macrophage subcluster in Col+nHA group versus Blank group.

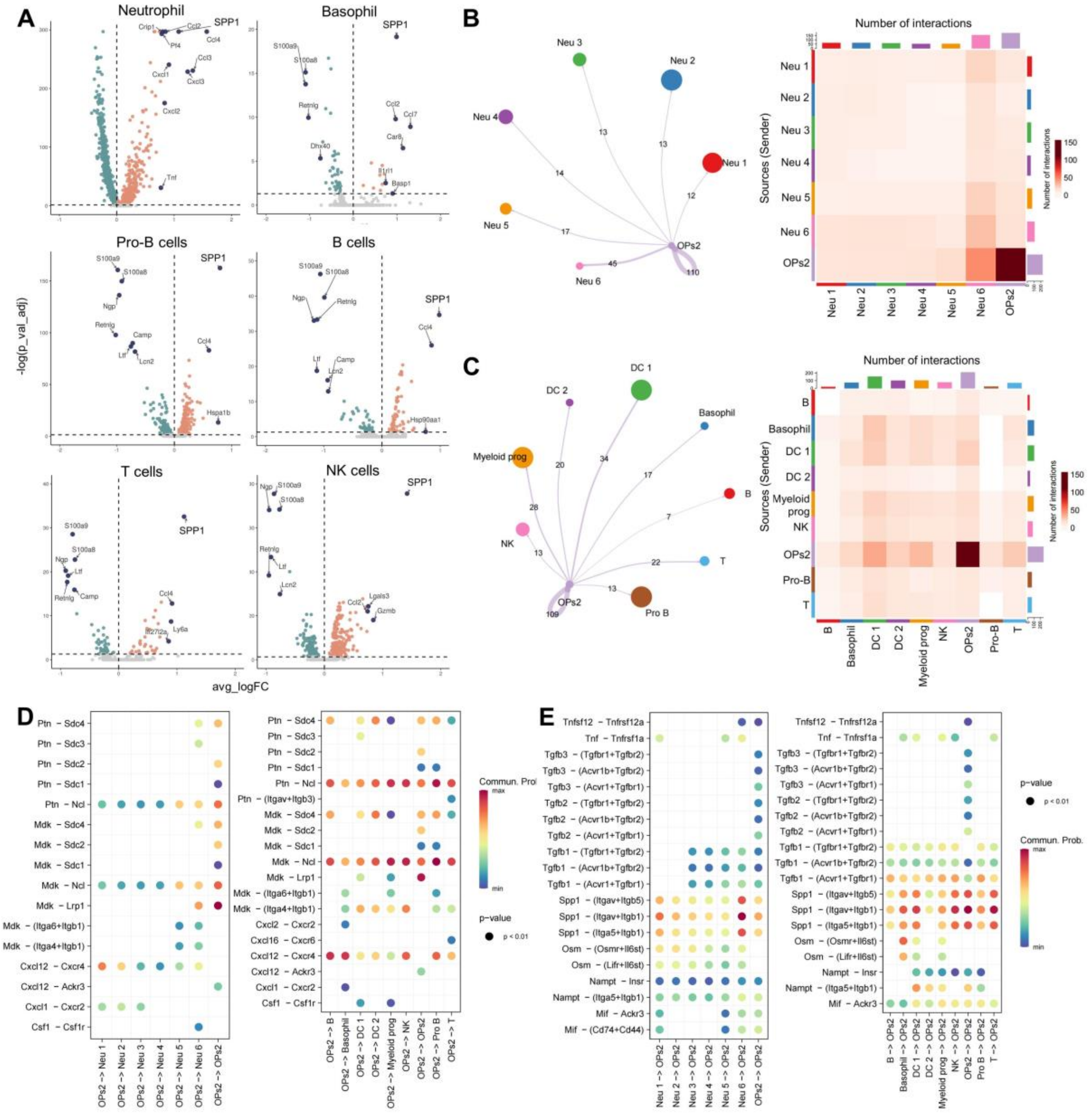


**Figure S11. Multiple immune cells performed high expression levels of *Spp1* and close interactions between OPs2 cells.** (A) Volcano plots displaying the down- and up-regulated genes in Col+nHA samples compared with the blank samples (from left to right). (B) Cellular interactions between OPs2 and neutrophil subclusters. Heatmap showing the number of interactions between OPs2 and neutrophil subclusters. (C) Cellular interactions between OPs2 and other immune cell clusters. Heatmap showing the number of interactions between OPs2 and immune cell clusters. (D) Unique ligand-receptor pairs between OPs2 and multiple immune cells. OPs2 functioned as the regulator. (E) Unique ligand-receptor pairs between OPs2 and multiple immune cells. OPs2 functioned as the receiver.

**Table S1.** Primer sequences used in qRT-PCR.

| Gene | Forward Primer (5' to 3') | Reverse Primer (3' to 5') |
|---|---|---|
| *GAPDH* | GAAGGTGAAGGTCGGAGTC | GAAGATGGTGATGGGATTTC |
| *MGP* | CCTCAGCAGAGATGGAGAGCTAA | GAGCGTTCTCGGATCCTCTCTT |
| *ALP* | ATGGGATGGGTGTCTCCACA | CCACGAAGGGGAACTTGTC |
| *OSX* | CCTCCTCAGCTCACCTTCTC | GTTGGGAGCCCAAATAGAAA |
| *RUNX2* | GAACCACAAGTGCGGTGCAA | ACTGCTTGCAGCCTTAAATGACT |
| *OCN* | ACACTCCTCGCCCTATTGGC | TGCTTGGACACAAAGGCTGC |
| *iNOS* | CTGGCAAGCCCAAGGTCTAT | TCCCCGCAAACATAGAGGTG |
| *IL-1β* | ATGATGGCTTATTACAGTGGCAA | GTCGGAGATTCGTAGCTGGA |
| *IL-6* | TGGCAGAAAACAACCTGAACC | TGGCTTGTTCCTCACTACTCTC |
| *IL-10* | TCAAGGCGCATGTGAACTCC | GATGTCAAACTCACTCATGGCT |
| *TNF-α* | CTGCACTTTGGAGTGATCGG | GGTTTGCTACAACATGGGCT |
| *TGF-β* | GGCCAGATCCTGTCCAAGC | GTGGGTTTCCACCATTAGCAC |
| *ARG-1* | CTCAAAGGGACAGCCACGAG | TAGGGATGTCAGCAAAGGGC |
| *CD206* | ATTCAGATATGCCAGGGCGA | CCTGCAATCCCGGTTCTCAT |
| *TRAP* | GACTGTGCAGATCCTGGGTG | GGTCAGAGAATACGTCCTCAAAG |
| *RANK* | AGATCGCTCCTCCATGTACCA | GCCTTGCCTGTATCACAAACTTT |
| *CTSK* | ACACCCACTGGGAGCTATG | GACAGGGGTACTTTGAGTCCA |
| *CTR* | CCTATCCAACAATAGAGCCCAAG | TGCATTCGGTCATAGCATTTGTA |
| *M-CSF* | TGGCGAGCAGGAGTATCAC | AGGTCTCCATCTGACTGTCAAT |
| *MDK* | CCTGCAACTGGAAGAAGGAG | CTGGCACTGAGCATTGTAGC |

**Table. Primer sequences used in qRT-PCR.** *GAPDH*: glyceraldehyde-3-phosphate dehydrogenase; *MGP*: matrix Gla protein; *ALP*: alkaline phosphatase; *OSX*: osterix; *RUNX2*: runt related transcription factor 2; *OCN*: osteocalcin; *iNOS*: inducible nitric oxide synthase; *IL-1β*: interleukin 1 beta; *IL-6*:interleukin 6; *IL-10*:interleukin 10; *TNF-α*: tumor necrosis factor alpha; *TGF-β*: transforming growth factor beta; *ARG-1*: arginase 1; *CD206*: CD206 molecule; *TRAP*: triiodothyronine receptor auxiliary protein; *RANK*: receptor activator of nuclear factor kappa; *CTSK*: cathepsin K; *CTR*: calcitonin receptor; *M-CSF*: macrophage colony-stimulating factor; *MDK*: midkine.
